# Maternal and early postnatal immune activation produce sex-specific effects on autism-like behaviors and neuroimmune function in mice

**DOI:** 10.1038/s41598-019-53294-z

**Published:** 2019-11-15

**Authors:** William A. Carlezon, Woori Kim, Galen Missig, Beate C. Finger, Samantha M. Landino, Abigail J. Alexander, Emery L. Mokler, James O. Robbins, Yan Li, Vadim Y. Bolshakov, Christopher J. McDougle, Kwang-Soo Kim

**Affiliations:** 1 Basic Neuroscience Division, Harvard Medical School, McLean Hospital, Belmont, MA USA; 2 Lurie Center for Autism, Harvard Medical School, Massachusetts General Hospital, Lexington, MA USA

**Keywords:** Neuroscience, Neuroimmunology

## Abstract

Increasing evidence suggests a role for inflammation in neuropsychiatric conditions including autism spectrum disorder (ASD), a neurodevelopmental syndrome with higher prevalence in males than females. Here we examined the effects of early-life immune system activation (EIA)—comprising regimens of prenatal, early postnatal, or combined (“two-hit”) immune activation—on the core behavioral features of ASD (decreased social interaction, increased repetitive behavior, and aberrant communication) in C57BL/6J mice. We treated timed-pregnant mice with polyinosinic:polycytidylic acid (Poly I:C) on gestational day 12.5 to produce maternal immune activation (MIA). Some offspring also received lipopolysaccharide (LPS) on postnatal day 9 to produce postnatal immune activation (PIA). EIA produced disruptions in social behavior and increases in repetitive behaviors that were larger in males than in females. Ultrasonic vocalizations (USVs) were altered in both sexes. Molecular studies revealed that EIA also produced prominent sex-specific changes in inflammation-related gene expression in the brain. Whereas both sexes showed increases in pro-inflammatory factors, as reflected by levels of mRNA and protein, expression of anti-inflammatory factors was decreased in males but increased in females. Our findings demonstrate that EIA can produce sex-specific behavioral effects and immune responses in the brain, and identify molecular processes that may contribute to resilience in females.

## Introduction

There is increasing evidence that the immune system is involved in neuropsychiatric illnesses^1^. There are higher rates of autism spectrum disorder (ASD) in families with a history of autoimmune disorders^2^, and maternal infection or fever during pregnancy increases the risk that offspring will develop ASD^3,4^. Furthermore, individuals with ASD often have higher levels of systemic pro-inflammatory cytokines^5^, and transcriptomic analysis of post-mortem brain samples reveals upregulation of immune response genes^6^. Such findings support the hypothesis that there is an immune-based subtype of ASD^7^ that accounts for some—though not all—cases of this condition.

Animal models involving early-life immune activation provide support for a causal role of immune dysregulation in ASD. While the contribution of genetics to ASD is well-established^8–10^, studies in laboratory animals demonstrate that environmental factors such as those that trigger early-life immune system activation (EIA) can be sufficient to cause phenotypes that resemble ASD or co-morbid conditions^11,12^. Immune system activation during pregnancy—maternal immune activation (MIA)—in rodents and non-human primates can result in offspring that exhibit three core behavioral features of ASD, including decreased social interaction, aberrant communication, and increased repetitive or focused behavior^13–15^. In a prototypical MIA regimen^13^, pregnant dams are treated on embryonic day 12.5 (E12.5) with polyinosinic:polycytidylic acid (Poly I:C)—a toll-like receptor-3 (TLR3) agonist that simulates an innate response to viral infection—which exposes offspring to immune challenge *in utero*. Similarly, postnatal immune activation (PIA) involving early developmental treatment of the offspring with lipopolysaccharide (LPS)—a TLR4 agonist that simulates an innate response to bacterial infection—can cause a variety of behavioral changes in mice that resemble features of ASD^16^.

The efficacy of the prototypical MIA (Poly I:C) regimen can be variable, particularly with respect to behavioral endpoints^17^. Baseline stress or differences in gut flora^18^ influence the efficacy of MIA regimens. Such factors may act as “disease primers”, by making an organism more susceptible to effects of environmental stressors or genetic mutations^19^. Indeed, peripubertal stress can reportedly unmask or exacerbate the behavioral and neurochemical consequences of MIA^20^. To further explore the possibility of accumulating effects, we developed a “two-hit” EIA regimen in mice that involves MIA (using Poly I:C) followed by PIA (using LPS). This paradigm enables studies of whether MIA can increase the susceptibility of offspring to the effects of a second immune challenge early in postnatal development. We have recently shown in male mice that this two-hit regimen can produce persistent alterations in sleep and epileptiform activity, resembling co-morbid medical conditions often seen in ASD^11^. It also alters the excitatory/inhibitory balance in projections from medial prefrontal cortex (mPFC) to basolateral amygdala (BLA), a circuit implicated in human autism^17^. Other groups have also described the effect of multiple “hits” on core behavioral features in mice^21^. Here, we used the two-hit approach to examine behavioral endpoints intended to reflect the core features of ASD: social interaction, repetitive behavior, and ultrasonic vocalizations. In parallel, we systematically examined the molecular consequences of these treatments, focusing on gene expression changes (both mRNA and protein levels) of pro-inflammatory genes, neuroinflammation marker genes, and anti-inflammatory genes in brain areas implicated in emotion and motivation. Considering previous reports of sex-dependent effects of both prenatal and postnatal immune activation^22,23^, we examined both sexes across behavioral and molecular endpoints.

## Methods

### Subjects and treatments

**Figure 1:**
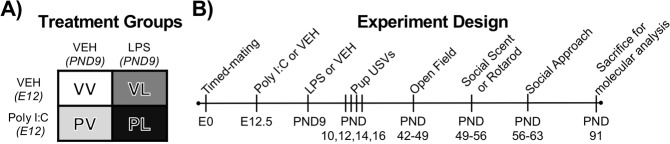
Depiction of (**A**) treatment groups and (**B**) study design.

All procedures were approved by McLean Hospital Institutional Animal Care and Use Committee and methods were performed in accordance with all relevant guidelines and regulations including the National Institutes of Health’s (NIH) Guide for the Care and Use of Laboratory Animals. Timed pregnancies in C57BL/6J mice (6–8 weeks, Jackson Laboratories, Bar Harbor, ME) were implemented by housing a female and male pair overnight, as described^11,17^. The following morning, pairs were separated and mid-day was considered embryonic day 0.5 (E0.5). Pregnancy was identified by weight gain and physical appearance. At E12.5 pregnant dams received intraperitoneal (IP) injections of 20 mg/kg Poly I:C (“P”) (Catalog #P9852, Sigma-Aldrich, St. Louis, MO, USA) or vehicle (“V”) (sterile phosphate-buffered saline [PBS]). Dams were monitored daily for parturition, following which litters were left undisturbed. On postnatal day 9 (PND9), male and female pups received subcutaneous (SC) injections of 10 mg/kg of LPS (“L”) from *Escherichia coli* 0111:B4 (Catalog #L3024, Sigma-Aldrich) or endotoxin-free saline vehicle (“V”), yielding 4 different treatment groups (VV, PV, VL, PL) (Fig. 1A). Mice were weaned at 3–4 weeks of age and placed into single-sex, single-treatment condition cages. All mice from each litter were used for analyses, with 3–5 litters used for each treatment group.

### Behavioral analyses

Male and female offspring were tested in a battery of behavioral tests (Fig. 1B), with males being evaluated in an additional assay (social scent USVs) not validated in females^24^. Behavioral testing was performed in multiple small cohorts, with cohorts tested in pup USVs, open field, social approach tests, or the rotarod test, or the social scent test.

#### Ultrasonic vocalizations

Pup ultrasonic vocalizations (USVs) were recorded on postnatal day 10 (PND10), PND12, PND14, and PND16. Pups were removed from their home cage and placed in a sound-attenuating, Styrofoam recording chamber, thereby subjecting them to isolation from their mothers. The USVs were recorded for 3 min at room temperature using an ultrasonic microphone (CM16/CMPA, Avisoft Bioacoustics, Berlin, Germany) positioned 17 cm above the pup. Calls were transmitted directly to a pre-amplifier (Avisoft Ultrasoundgate 416H, Avisoft) connected to a computer that stored the sonograms. Avisoft SASLab Pro software (Version 5.1) was used for quantitative analysis of USV metrics (call count, frequency, duration). After 3 min of recording, pups were relocated to a new cage containing bedding, a method used to ensure all mice in the home cage were tested without marking them. The new cage was covered with a filter-top and placed on a heating pad. The acoustic chamber was cleaned with ethanol between trials.

#### Open field test

During postnatal week 6 (PND42–49), mice were removed from their home cage and placed in the center of the open field chamber (44 cm length × 44 cm width × 30 cm height), located in an adjacent room. Automated movement tracking and analysis in the open field chamber were conducted using Noldus Ethovision-XT 7.1 software. Endpoints included total distance traveled and the number of entries and time spent in the center of the open field (14 cm × 14 cm). Movement tracking began immediately upon placement in the Open Field and continued for 10 min. Following testing, the mice were re-located to a new cage, and the apparatus was cleaned with ethanol between each trial.

#### Rotarod test

During postnatal week 7 (PND49–56), mice were placed on an accelerating mouse rotarod (337500 series, TSE Systems). The rotarod accelerated from 4–40 RPM over the course of 5 min and the latency for the mouse to fall was automatically quantified. Mice were run on 3 consecutive trials with a 5-min inter-trial interval each day for 3 days. If a mouse fell within the first 10 sec of a trial, it was considered a false start and the mouse was re-tested.

#### Social scent test

During postnatal week 7 (PND49–56), male mice were removed from their home cage and placed in an empty clean cage in dim red light. Following a brief 2-min habituation, a cotton swab moistened with urine from a female mouse in estrus was placed in a 1-mL pipette and suspended from a metal grid on top of the cage. An observer blinded to treatment condition measured the time spent investigating the swab over the 5-min trial. The ultrasonic microphone was positioned above the cage to record USVs, which were scored manually using Avisoft SASLab Pro software. Urine was collected the same day from adult female mice determined to be in estrus via vaginal cytology.

#### Social approach test

During postnatal week 8 (PND56–63), mice were tested in the social approach test^25,26^. This test consisted of two, 3-min sessions in an enclosed arena (44 cm length × 44 cm width × 30 cm height) illuminated by dim red light. In Session 1, mice were placed in an arena with an empty metal wire cage (10 cm length × 8 cm width × 6 cm height) and time spent investigating the cage was measured. In Session 2, mice were placed in the same arena with an age-matched, sex-matched conspecific mouse in the metal wire cage. After completion of both sessions, the mice were re-located to a new cage, and the chamber was cleaned with ethanol wipes. A different conspecific mouse was used with each animal tested within a cage and stimulus mice were re-used across multiple experimental animals but were balanced across treatment groups. Data were analyzed with Noldus Ethovision-XT 7.1 software.

### Molecular analyses

#### Quantitative real-time reverse transcriptase polymerase chain reaction (qRT-PCR)

A subset of mice tested behaviorally (n ≥ 5 per group) were selected randomly at 13 weeks of age and killed by rapid decapitation. Brains were hemisected with a midline sagittal cut; the left hemisphere was used to analyze mRNA and the right hemisphere was used to analyze protein (below). Regions including medial prefrontal cortex (mPFC), amygdala (AMG), hippocampus (HP), and thalamus (TM) were rapidly dissected and stored at −80 °C until use. For qRT-PCR^27^, total RNA was isolated using GeneJET RNA Purification Kits (Thermo Fisher Scientific, Waltham, MA) according to the manufacturer’s protocols, and cDNA synthesis was performed with an Invitrogen Superscript cDNA Synthesis Kits (Thermo Fisher Scientific). In a mixture with 2X SsoAdvanced Universal SYBR Green Supermix (Bio-Rad, Hercules, CA), qRT-PCR was run on the CFX Connet^TM^ Real-Time System (Bio-Rad) in a volume of 20 µl, with 20 µl of forward and reverse primers (100 ng/µl each) and 1 µl of cDNA sample (Table 1). Expression levels of target genes for each sample were quantified using the 2^∆∆CT^ method (*CT* of target gene – *CT* of GAPDH [glyceraldehyde-3-phosphate dehydrogenase]). For each target gene, 2^∆∆CT^ values were normalized to the VV group, and statistical analyses used the normalized values.

**Table 1:** Primers used for qRT-PCR.

Gene	Forward Primer	Reverse Primer
TNFα	TCCCAGGTTCTCTTCAAGGGA	GGTGAGGAGCACGTAGTCGG
iNOS	AACAGAGCCCTCAGCAGCATCCAT	CCAGGTGTTCCCCAGGCAGGTAG
IL-6	GTTCTCTGGGAAATCGTGGA	TGTACTCCAGGTAGCTATGG
IL-1β	TTGACGGACCCCAAAAGATG	AGAAGGTGCTCATGTCCTCA
Iba-1	GGATTTGCAGGGAGGAAAAG	TGGGATCATCGAGGAATTG
GFAP	TACCATGCCACGCTTCTCCTTGTC	ACGCTCCGCTCGCCCGTGTCTCCT
TSPO	TGGTATGCTAGCTTGCAGAAACC	TCCCAGCTCTTTCCAGACTATGT
IL-10	CCTGGCTCAGCACTGCTAT	GCTCTTATTTTCACAGGGGAGAA
TGF-β1	GAAGCAGTGCCCGAACCCCC	GTGCAGGTGCTGGGCCCTTT

Forward and reverse primers used for qPCR analysis.

#### Protein (Western) immunoblotting

Protein samples were purified from the dissected brain tissue samples by homogenization and sonication in cell lysis buffer (100 mM Tris-HCl (pH 7.5), 10 mM EDTA, 10 mM EGTA, 1% SDS, 20 mM NaCl (Sigma, St. Louis, MO) containing 1 mM PMSF, protease inhibitor cocktail, and phosphatase inhibitor cocktail (Thermo Fisher Scientific). Lysates were centrifuged at 14,000 rpm for 30 min at 4 °C, and supernatants were collected and stored at −80 °C before use. Protein amounts were determined using Bio-Rad Protein Assay Dye (Bio-Rad, Hercules, CA) according to the manufacturer’s protocol, which is based on Bradford method. Equal amounts of total protein (10 µg) were analyzed by Western blot with the following primary antibodies (Abcam, Cambridge, UK): IL-6 (ab9234; 1:2,500), Iba-1 (ab5076; 1:2,500), GFAP (ab10062; 1:2,500), TSPO (ab109497; 1:1,000), IL-10 (ab192269; 1:2,500), TGF-β1 (ab92486; 1:2,500), and β-actin (ab8227; 1:5,000). Immune reactive bands were developed with Novex^®^ Chemiluminescent Substrate (Invitrogen, Carlsbad, CA) and visualized by ChemiDoc^TM^ XRS+ (Bio-Rad) with Image Lab^TM^ Software (Version 6.0.1). Experiments were performed in duplicate. Quantification of immunoreactive bands was expressed as a ratio against β-actin using ImageJ software (NIH, Bethesda, MD). For full-length western blots of each protein analyzed see Supplementary Information.

#### Enzyme-linked immunosorbent assay (ELISA)

Cytokine analysis for IL-1β was performed using a sandwich ELISA kit (#503501 BioLegend, San Diego, CA) according to the manufacturer’s protocol. Serially diluted standards (recombinant mouse IL-1β) and equal amounts of protein samples quantified by Bradford assay were loaded and incubated in a plate coated with capture antibodies (purified anti-mouse/rat IL-1β (1:100)), followed by incubation with detection antibodies (biotin anti-mouse IL-1β (1:200)). Absorbance was detected using an ELISA reader (BioTek, Winooski, VT) at 450 nm. Samples were run in triplicate, and experiments were performed in duplicate.

### Statistical analyses

Data for each sex were analyzed separately, given existing evidence that there are profound sex differences in EIA effects in laboratory animals^28^ and in the prevalence of ASDs in humans^29^, both of which point to *a priori* differences in the distribution of traits regulating behavior and gene expression in males and females^30^. Two-way ANOVAs (prenatal x postnatal treatment) was used for the social approach and open field tests. We previously reported data from a subset of the male mice used in these behavioral tests within a study that focused primarily on the electrophysiological consequences of perinatal immune activation^17^. Three-way ANOVAs (prenatal x postnatal treatment x day (repeated)) was used for rotarod or pup USVs. The Kruskal-Wallis test was used for analysis of social scent USVs due to a non-normal distribution. Molecular data were compared using two-way ANOVAs (prenatal × postnatal treatment) for each gene/protein in each region. *Post hoc* comparisons with Bonferroni’s multiple comparison test were used to compare all groups to VV and control for multiple comparisons. Pearson linear correlation analysis was used for the relationship between mRNA and protein expression levels. Analyses were performed with GraphPad Prism 8.0 or SPSS 24.0; exact *P* values are provided unless *P* < 0.001.

## Results

### Communication-like Behavior

**Figure 2:**
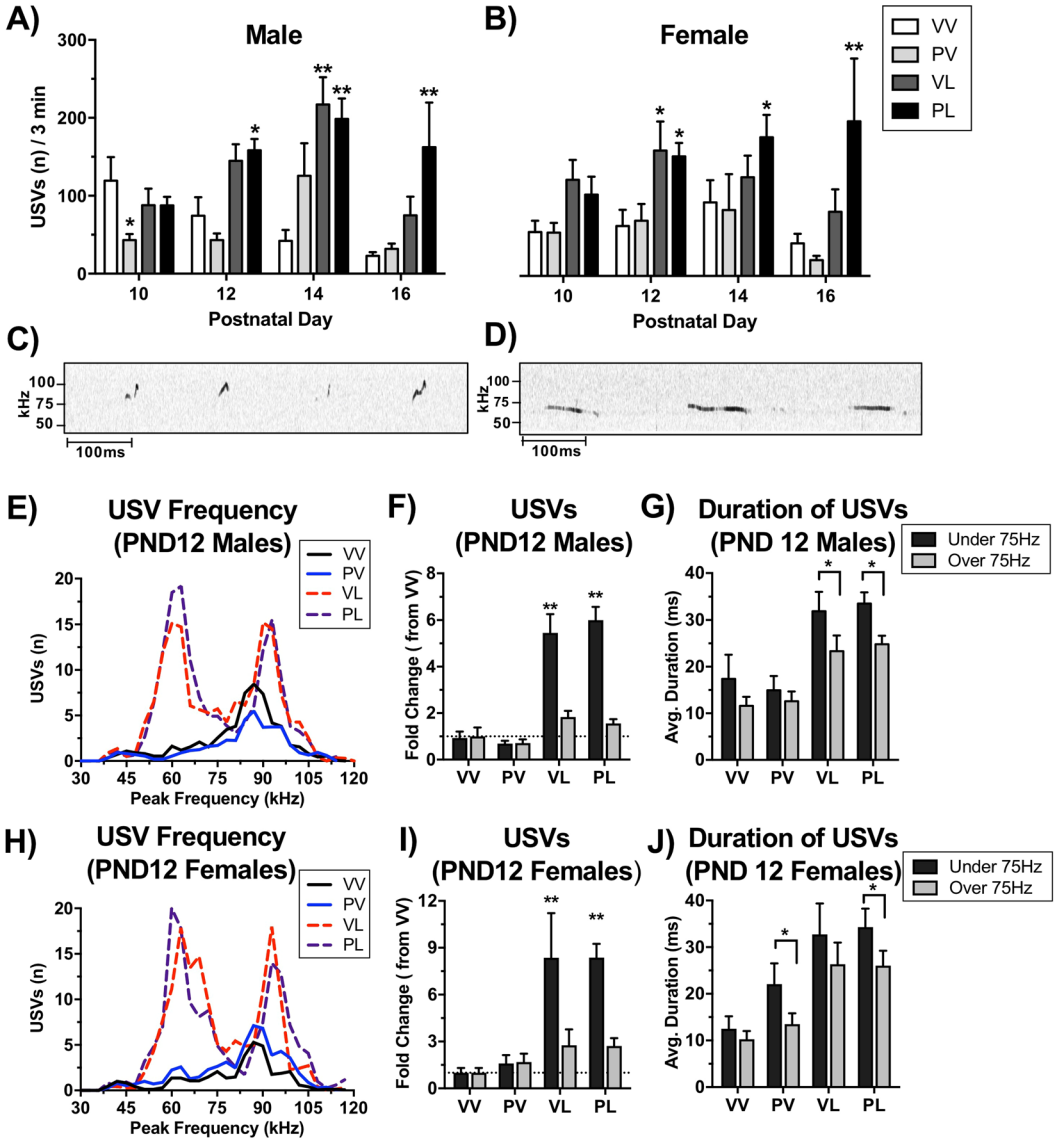
Treatment effects on mouse pup ultrasonic vocalizations (USVs) and call microstructure. Maternal separation was used to evoke USVs in male (**A**) and female (**B**) mouse pups on postnatal days (PND) 10, 12, 14, and 16. Total number of USVs per 3 min were quantified; data are expressed as Mean + SEM *PND 10* (*Male n* = *VV: 14*, *PV: 22*, *VL: 15*, *PL: 23*) (*Female n* = *VV: 18*, *PV:18*, *VL: 8*, *PL: 12*), *PND12* (*Male n* = *VV: 13*, *PV: 21*, *VL: 15*, *PL: 23*) (*Male n* = *VV: 14*, *PV: 22*, *VL: 15*, *PL: 23*) (*Female n* = *VV: 16*, *PV: 16*, *VL: 8*, *PL: 12*), *PND14* (*Male n* = *VV: 8*, *PV: 8*, *VL: 14*, *PL: 25*) (*Female n* = *VV: 11*, *PV: 6*, *VL: 7*, *PL: 17*) *PND16* (*Male n* = *VV: 15 PV: 22*, *VL: 10*, *PL: 7*) (*Female n* = *VV: 18*, *PV: 18*, *VL: 6*, *PL: 4*). Representative tracings on Day 12 from mice that received the control (**C**) and “two-hit” (**D**) treatments revealed dramatic differences in USVs. Detailed analyses revealed major differences in call numbers, patterns, and durations in males (**E–G**) and females (**H–J**). *P < 0.05, **P < 0.01 compared to VV group, Bonferroni’s tests.

**Figure 3:**
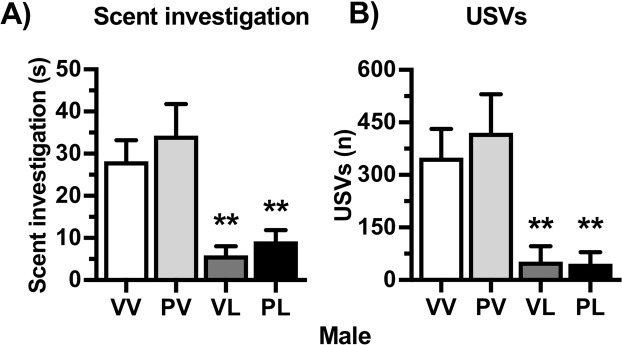
Treatment effects on social scent-evoked USVs (Mean + SEM; *n* = VV: 25, PV: 13, VL: 15, PL: 15) in juvenile (7-week old) male mice. Postnatal LPS significantly reduced (**A**) investigation of the scent (urine from an estrous female mouse) and (**B**) USVs. **P < 0.01 compared to VV, Bonferroni’s tests.

EIA produced alterations in both call number and call characteristics that differed between prenatal and postnatal immune activation (Fig. 2A,B). Prenatal Poly I:C (PV) treatment reduced the number of USVs (F(3,239) = 3.16, *P* = 0.025), with a significant reduction (compared to VV controls) on Day 10 (*P* < 0.05, Bonferroni’s multiple comparison test) in males, with no significant effects of prenatal treatment in females. In contrast, postnatal LPS treatment led to an increase in USVs in both males (F(3,239) = 5.71, *P* = 0.001) and females (F(3,179) = 14.78, *P* < 0.001) that emerged over days. In males, USVs were significantly increased (compared to VV controls) in PL mice on PND12 (*P* < 0.01), and in both VL and PL mice on PND14 (*P’s* < 0.01). In females, USVs were significantly increased (compared to VV controls) in both VL and PL mice on PND12 (*P* < 0.05 and *P* < 0.01, respectively) and PL mice on PND14 (*P* < 0.05). For both sexes, the combination of prenatal and postnatal immune activation (PL) prolonged the increase of USVs emitted through PND16 (*P’s* < 0.01). Inspection of call characteristics in control and LPS-treated mice revealed striking qualitative differences in their microstructure (Fig. 2C,D). To compare call characteristics in further detail, we analyzed peak frequency and duration of calls on PND12. Whereas the microstructure of calls from VV and PV mice showed normal distributions with peak frequency of ~90 kHz, calls from both VL and PL mice (i.e., those that received either LPS exposure regimen) showed bimodal distributions, with peaks at ~60 kHz and ~90 kHz (Fig. 2E,H). The majority of the increases in USVs in LPS-treated mice was due to an increase in > 75 kHz USVs in both males (F(1,127) = 105.21, *P* < 0.001) (Fig. 2F) and females (F(1,98) = 28.62, *P* < 0.001) (Fig. 2I**)**. In general, > 75 kHz USVs were longer in duration than < 75 kHz USVs in both males (F(1,127) = 12.95, *P* < 0.001) and females (F(1,98) = 6.25, *P* = 0.014), and this effect was enhanced in LPS-treated mice (Males: F(1,127) = 9.72, *P* = 0.002; Females: F(1,98) = 13.48, *P* < 0.001) (Fig. 2G,J). These results indicate that postnatal LPS (single- or double-hit) alters the number and microstructural characteristics of USVs in both sexes. To determine if these alterations in putative communication-like behavior continues into adulthood, we measured USVs evoked by a social scent in young-adult male mice (7 weeks of age). Compared to VV controls, LPS-treated male mice presented with a cotton swab laced with urine from an estrus female mouse spent less time investigating the swab (H = 22.09, *P* < 0.001; VL and PL, *P’s* < 0.01) (Fig. 3A) and emitted fewer USVs (H = 24.25, *P* < 0.001; VL and PL, *P’s* < 0.01) (Fig. 3B**)**. Considered together, these data suggest that postnatal LPS can produce significant alterations in communication-like behaviors that persist throughout the lifespan in both sexes, whereas prenatal Poly I:C effects are more transient, at least under the conditions tested.

### Social and anxiety-like behaviors

**Figure 4:**
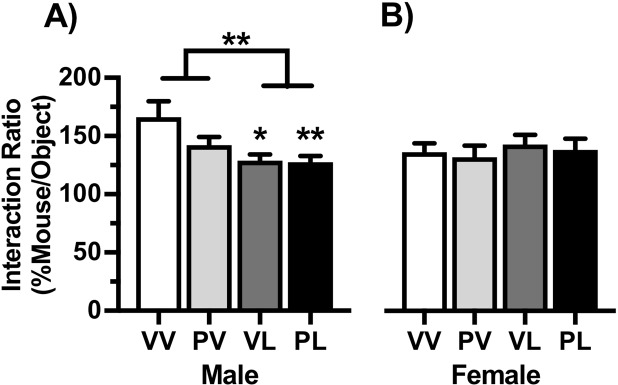
Treatment effects on social behavior. Data are expressed as Mean + SEM (*Male n* = *VV: 12*, *PV: 17*, *VL: 11*, *PL: 18*) (*Female n* = *VV: 18*, *PV:18*, *VL: 11*, *PL: 11*) ratio of the time spent investigating an empty cage to the time spent investigating an age- and sex-matched conspecific mouse within the cage during 150-sec test sessions. Postnatal LPS significantly reduced the interaction ratio in males (**A**) but not females (**B**). *P < 0.05, **P < 0.01 compared to VV, Bonferroni’s tests.

**Figure 5:**
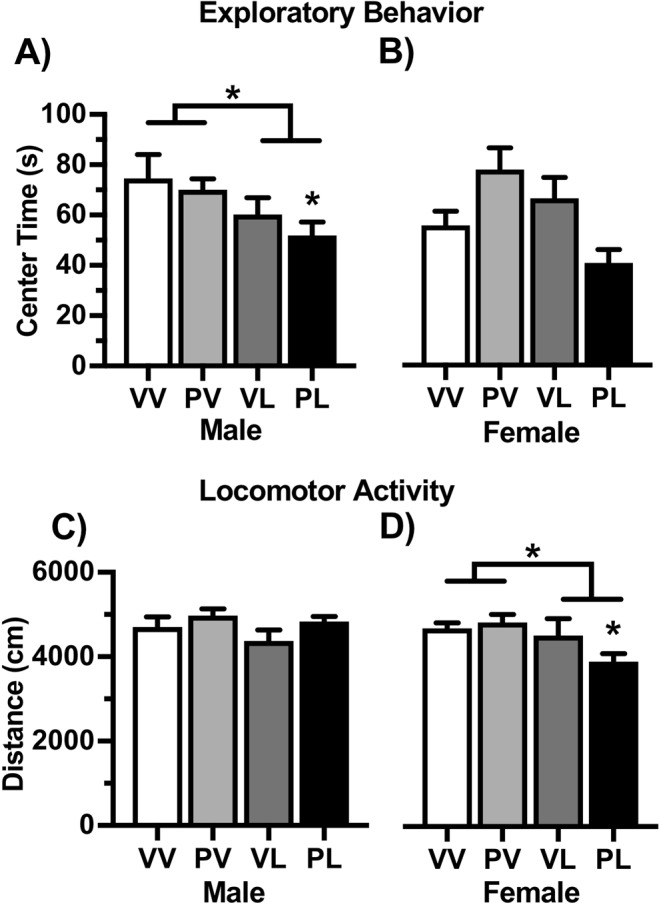
Treatment effects on behavior in an open field. Data are expressed as Mean + SEM (*Male n* = *VV: 13*, *PV: 18*, *VL: 11*, *PL: 17*) (*Female n* = *VV: 17*, *PV:17*, *VL: 10*, *PL: 10*). Postnatal LPS significantly reduced center time in males, and effect that was greatest in the PL (“two-hit”) condition, without producing significant effects in females (**A**). Locomotor activity was not significantly altered in males, but was significantly decreased in females following LPS, especially when combined with Poly I:C. (**B**). *P < 0.05, **P < 0.01 compared to VV, Bonferroni’s tests.

Social approach data are expressed as a ratio of the time spent investigating an empty cage during Session 1 to the time spent investigating an age- and sex-matched conspecific mouse within the cage during Session 2. Compared to VV controls, the interaction ratios of LPS-treated mice were significantly lower in males (F(1,54) = 10.16, *P* = 0.003; VL and PL, *P’s* < 0.01) but unchanged in females (Fig. 4), suggesting male-specific deficits in this behavioral domain. When anxiety-like behavior was assessed in the open field test, center time was significantly lower in LPS-treated males (main effect: F(1,55) = 6.25, *P* = 0.015), with a significant reduction versus controls only in in those given the “two-hit” (PL) treatment (*P* < 0.05), whereas there were no significant effects in females (Fig. 5A). There were no statistically significant effects on overall locomotor activity levels in males (Fig. 5B**)**, however there was a significant reduction in females (main effect: (1,49) = 6.33, *P* = 0.015) that was more pronounced with PL treatment (*P* < 0.05). These data indicate that EIA produces alterations in social and anxiety-like behaviors that are most prominent in males.

### Repetitive behaviors

**Figure 6:**
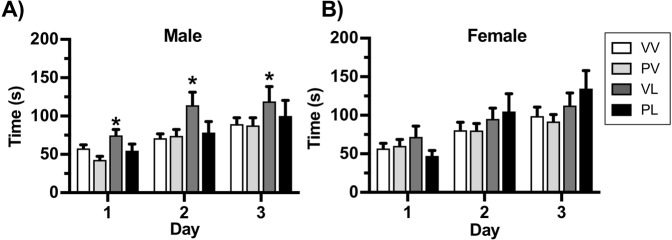
Treatment effects on performance on the rotarod, expressed as Mean + SEM time before falling (*Male n* = *VV: 25*, *PV: 20*, *VL: 11*, *PL: 9*) (*Female n* = *VV: 26*, *PV:18*, *VL: 16*, *PL: 10*). Postnatal LPS significantly increased the time before falling in males on each of the 3 test days (**A**) without producing significant effects in females (**B**). *P < 0.05, **P < 0.01 compared to VV, Bonferroni’s tests.

Although the rotarod test is typically used to evaluate motor coordination, it was recently reported that mice with genetic mutations related to ASD perform better in the task, which was interpreted as reflecting increased repetitive behavior^31^. Mice were placed on an accelerating rotarod and assessed for latency to fall for three trials on three consecutive days. Overall, performance improved over the three days, as indicated by longer trials in both males (F(1,61) = 50.16, *P* < 0.001) (Fig. 6A) and females F(1,61) = 59.25, *P* < 0.001) (Fig. 6A). In males there was an effect of postnatal LPS treatment (F(1,61) = 5.18, *P* = 0.027) that was attributable to the VL-treated mice (Days 1–3, *P’s* < 0.05), with no significant treatment effects in females. These data suggest that postnatal LPS may cause modest increases in repetitive behaviors that are most prominent in males, and raise the possibility that in the case of this specific domain, MIA (with Poly I:C) can produce protective-like effects.

### Expression of pro-inflammatory markers

**Figure 7:**
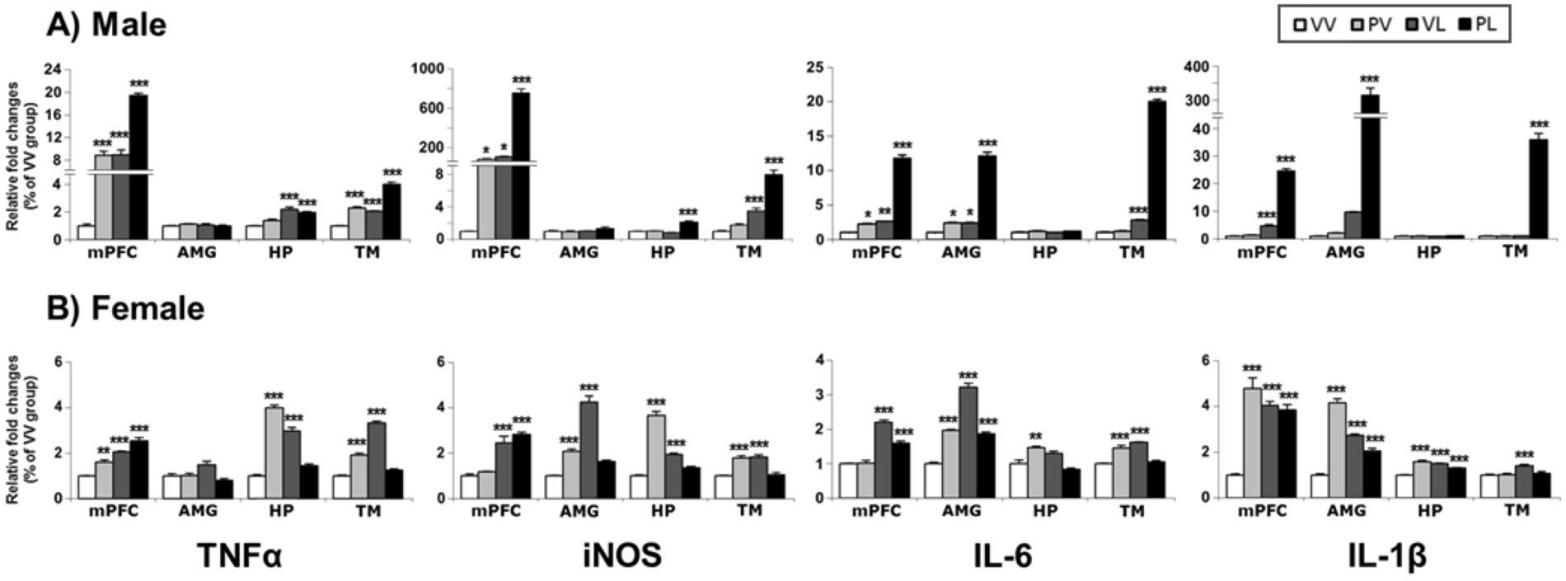
Levels of mRNA expression for pro-inflammatory markers in the brains of (**A**) male and (**B**) female mice following perinatal immune activation, expressed as Mean + SEM (*n* = 5/group) of duplicate experiments. *P < 0.05, **P < 0.01, ***P < 0.001 compared to VV, Bonferroni’s *post-hoc* test. TNFα, tumor necrosis factor alpha; iNOS, inducible nitric oxide synthase; IL-6, interleukin-6; IL-1β, interleukin-1 beta; mPFC, prefrontal cortex; AMG, amygdala, HP, hippocampus; TM, thalamus.

We examined EIA effects on expression (as reflected by mRNA levels) on a restricted set of pro-inflammatory genes (e.g., tumor necrosis factor alpha [TNFα], inducible nitric oxide synthase [iNOS], interleukin-6 [IL-6] and interleukin-1 beta [IL-1β]) in each of four brain areas (mPFC, AMG, HP, and TM). The results of the ANOVAs are provided in Table 2. In general, all of these markers were elevated in both males and females, although there were some qualitative differences. In males (Fig. 7A), general themes were that the two-hit (PL) effects were largest, the Poly I:C (PV) effects were most variable, and some regions (e.g., HP) were less reliably affected. In females (Fig. 7B), the same general pattern of effects was observed, except all regions were more reliably affected.

**Table 2:** Pro-inflammatory markers, changes in mRNA and protein levels, and correlation between mRNA and protein expressions.

Sex	Endpoint	Marker	Region	Analysis				*Post-hoc* compared to VV	Protein-mRNA
				Main effect: postnatal		Interaction			
				Statistic (df)	*P* value	Statistic (df)	*P* value		
Male	mRNA	TNFα	mPFC	F(1,16) = 228.87	<0.0001	F(1,16) = 4.19	*ns*	PV, VL, PL	
			AMY	F(1,16) = 0.12	*ns*	F(1,16) = 2.09	*ns*		
			HP	F(1,16) = 84.02	<0.0001	F(1,16) = 11.11	0.0042	VL, PL	
			TM	F(1,16) = 208.74	<0.0001	F(1,16) = 11.00	0.0044	PV, VL, PL	
		iNOS	mPFC	F(1,16) = 329.51	<0.0001	F(1,16) = 172.74	<0.0001	PV, VL, PL	
			AMY	F(1,16) = 3.43	*ns*	F(1,16) = 3.03	*ns*		
			HP	F(1,16) = 36.01	<0.0001	F(1,16) = 64.70	<0.0001	PL	
			TM	F(1,16) = 161.51	<0.0001	F(1,16) = 29.03	<0.0001	VL, PL	
		IL-6	mPFC	F(1,16) = 476.19	<0.0001	F(1,16) = 241.79	<0.0001	PV, VL, PL	
			AMY	F(1,16) = 331.12	<0.0001	F(1,16) = 185.11	<0.0001	PV, VL, PL	
			HP	F(1,16) = 0.22	*ns*	F(1,16) = 0.03	*ns*		
			TM	F(1,16) = 4761.16	<0.0001	F(1,16) = 3270.32	<0.0001	VL, PL	
		IL-1β	mPFC	F(1,16) = 742.96	<0.0001	F(1,16) = 389.32	<0.0001	VL, PL	
			AMY	F(1,16) = 227.53	<0.0001	F(1,16) = 203.69	<0.0001	PL	
			HP	F(1,16) = 0.90	*ns*	F(1,16) = 1.66	*ns*		
			TM	F(1,16) = 219.60	<0.0001	F(1,16) = 217.58	<0.0001	PL	
Female	mRNA	TNFα	mPFC	F(1,16) = 126.28	<0.0001	F(1,16) = 0.36	*ns*	PV, VL, PL	
			AMY	F(1,16) = 1.37	*ns*	F(1,16) = 8.96	0.0086		
			HP	F(1,16) = 6.64	0.0203	F(1,16) = 435.01	<0.0001	PV, VL, PL	
			TM	F(1,16) = 126.54	<0.0001	F(1,16) = 407.69	<0.0001	PV, VL	
		iNOS	mPFC	F(1,16) = 101.98	<0.0001	F(1,16) = 0.36	*ns*	VL, PL	
			AMY	F(1,16) = 88.52	<0.0001	F(1,16) = 154.85	<0.0001	PV, VL	
			HP	F(1,16) = 47.78	<0.0001	F(1,16) = 278.79	<0.0001	PV, VL	
			TM	F(1,16) = 0.36	*ns*	F(1,16) = 81.59	<0.0001	PV, VL	
		IL-6	mPFC	F(1,16) = 217.47	<0.0001	F(1,16) = 25.92	0.0001	VL, PL	
			AMY	F(1,16) = 224.38	<0.0001	F(1,16) = 271.55	<0.0001	PV, VL, PL	
			HP	F(1,16) = 5.06	0.0389	F(1,16) = 41.85	<0.0001	PV	
			TM	F(1,16) = 6.02	0.026	F(1,16) = 149.25	<0.0001	PV, VL	
		IL-1β	mPFC	F(1,16) = 14.05	0.0018	F(1,16) = 49.06	<0.0001	PV, VL, PL	
			AMY	F(1,16) = 2.29	*ns*	F(1,16) = 248.65	<0.0001	PV, VL, PL	
			HP	F(1,16) = 9.36	0.0075	F(1,16) = 112.82	<0.0001	PV, VL, PL	
			TM	F(1,16) = 15.34	0.0012	F(1,16) = 9.03	0.0084	VL	
Male	protein	IL-6	mPFC	F(1,8) = 1.70	*ns*	F(1,8) = 1.47	*ns*		R^2^ = 0.0458, *ns*
			AMY	F(1,8) = 7.76	0.0237	F(1,8) = 0.03	*ns*	PL	R^2^ = 0.2755, *ns*
			HP	F(1,8) = 5.97	0.0403	F(1,8) = 0.34	*ns*		R^2^ = 0.001, *ns*
			TM	F(1,8) = 116.22	<0.0001	F(1,8) = 3.72	*ns*	VL, PL	R^2^ = 0.7313, *P* = 0.0004
		IL-1β	mPFC	F(1,8) = 289.30	<0.0001	F(1,8) = 68.24	<0.0001	VL, PL	R^2^ = 0.9592, *P* < 0.0001
			AMY	F(1,8) = 349.76	<0.0001	F(1,8) = 135.26	<0.0001	VL, PL	R^2^ = 0.9343, *P* < 0.0001
			HP	F(1,8) = 0.62	*ns*	F(1,8) = 0.90	*ns*		R^2^ = 0.0179, *ns*
			TM	F(1,8) = 76.01	<0.0001	F(1,8) = 77.07	<0.0001	PL	R^2^ = 0.9541, *P* < 0.0001
Female	protein	IL-6	mPFC	F(1,8) = 1.36	*ns*	F(1,8) = 0.29	*ns*		R^2^ = 0.3689, *P* = 0.0362
			AMY	F(1,8) = 21.02	0.0018	F(1,8) = 13.50	0.0063	PV, VL, PL	R^2^ = 0.4773, *P* = 0.0129
			HP	F(1,8) = 17.71	0.003	F(1,8) = 0.01	*ns*		R^2^ = 0.062, *ns*
			TM	F(1,8) = 1.95	*ns*	F(1,8) = 0.14	*ns*		R^2^ = 0.016, *ns*
		IL-1β	mPFC	F(1,8) = 0.11	*ns*	F(1,8) = 9.41	0.0154	PV	R^2^ = 0.4386, *P* = 0.019
			AMY	F(1,8) = 0.00	*ns*	F(1,8) = 23.59	0.0013		R^2^ = 0.7228, *P* = 0.0005
			HP	F(1,8) = 1.24	*ns*	F(1,8) = 1.99	*ns*		R^2^ = 0.2616, *ns*
			TM	F(1,8) = 12.03	0.0085	F(1,8) = 1.67	*ns*		R^2^ = 0.4137, *P* = 0.0241

Statistical analysis of pro-inflammatory mRNA and protein markers by brain region for males and females. *ns*, not significant; VV, Vehicle-Vehicle; PV, Poly I:C-Vehicle; VL, Vehicle-LPS; PL, Poly I:C-LPS.

**Figure 8:**
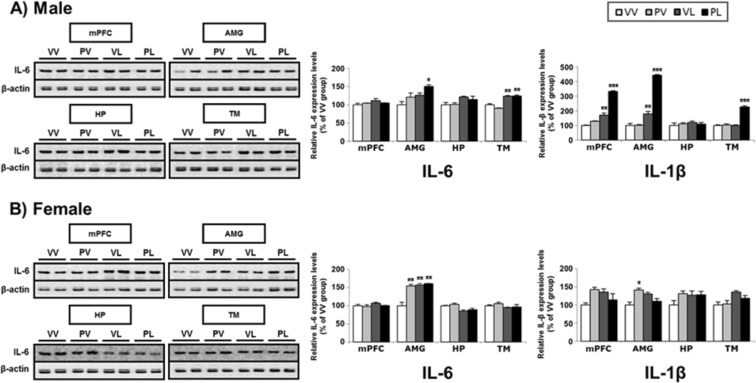
Representative western blots and levels of protein expression for pro-inflammatory markers (and β-actin control) in the brains of (**A**) male and (**B**) female mice following perinatal immune activation, expressed as Mean + SEM (*n* = 3/group) of triplicate experiments. IL-6 data is from western blot and IL-1β is from ELISA. Images of western blots are cropped selections from full length western blots found in supplementary information. *P < 0.05, **P < 0.01, ***P < 0.001 compared to VV, Bonferroni’s *post-hoc* test.

In parallel, we examined expression of the proteins encoded by these pro-inflammatory mRNAs (Table 2). Only data for IL-6 and IL-1β are presented, because the antibodies for TNFα and iNOS yielded weak and unreliable signals over a broad range of conditions. In general, there is correspondence between mRNA levels and protein expression for both sexes, with expression of these markers increasing in both sexes, and with the largest changes in AMG and smallest changes in HP (Fig. 8A,B). There was a small number of cases where significant elevations in mRNA levels were not accompanied by corresponding significant elevations in protein levels (for males, IL-6 in mPFC and HP, and IL-1β in AMG; for females, IL-6 in the mPFC and TM, and IL-1β in mPFC and HP), suggesting post-translational modifications. Correlation analysis revealed that IL-6 mRNA and protein level were correlated only in TM (R^2^ = 0.7313, *P* = 0.0004) of males and in mPFC (R^2^ = 0.3689, *P* = 0.0362) and AMG (R^2^ = 0.4773, *P* = 0.435) of females (Suppl. Fig. S1A). In case of IL-1β, both males and females showed positive correlations between mRNA and protein levels except for HP (Suppl. Fig. S1B).

### Expression of neuroinflammation markers

**Figure 9:**
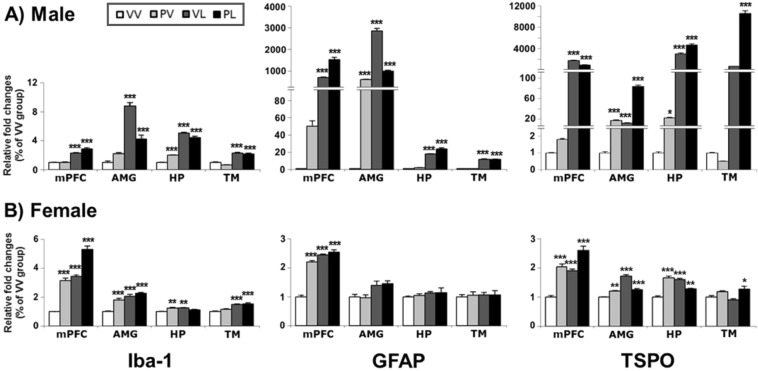
Levels of mRNA expression for neuroinflammation markers in the brains of (**A**) male and (**B**) female mice following perinatal immune activation, expressed as Mean + SEM (*n* = 5/group) of duplicate experiments. *P < 0.05, **P < 0.01, ***P < 0.001 compared to VV, Bonferroni’s *post-hoc* test. Iba-1, ionized calcium-binding adaptor molecule 1; GFAP, glial fibrillary acidic protein; TSPO, translocator protein.

**Figure 10:**
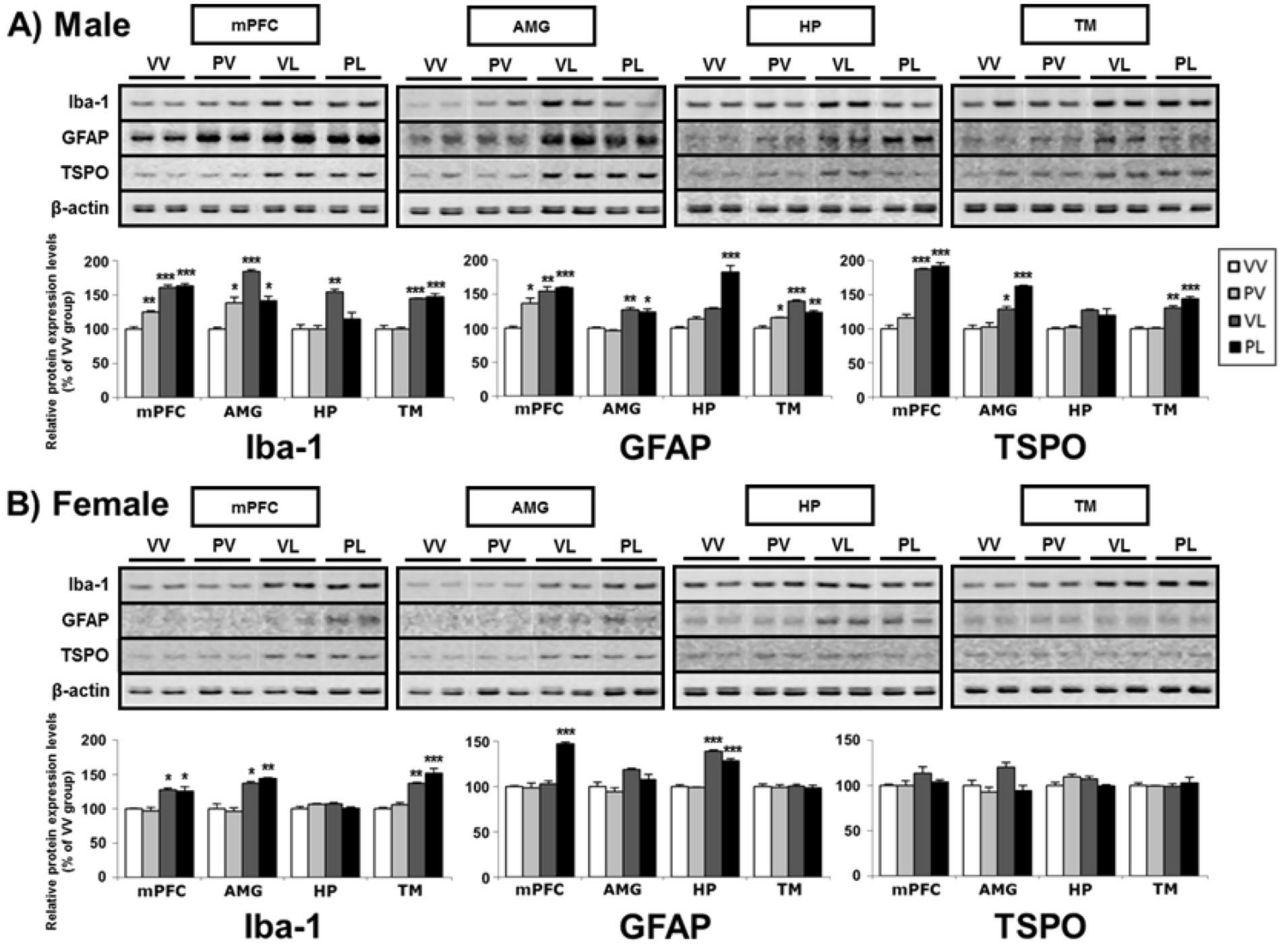
Representative western blots and levels of protein expression for neuroinflammation markers (and β-actin control) in the brains of (**A**) male and (**B**) female mice following perinatal immune activation, expressed as Mean + SEM (*n* = 3/group) of triplicate experiments. Images of western blots are cropped selections from full length western blots found in supplementary information. *P < 0.05, **P < 0.01, ***P < 0.001 compared to VV, Bonferroni’s *post-hoc* test.

We also examined mRNA and protein levels of neuroinflammation markers (e.g., ionized calcium-binding adaptor molecule 1 [Iba-1], glial fibrillary acidic protein [GFAP] and translocator protein [TSPO] (Table 3). As was the case with the proinflammatory markers, we observed broad increases in the levels of mRNA (Fig. 9) and protein (Fig. 10) for these markers in both sexes. Increases in mRNA levels tended be several-fold larger in males than in females, although the magnitude of the changes in protein was similar in both sexes. In general the effects were largest in groups that received LPS, either alone or together with Poly I:C (i.e., VL or PL), although there was a single instance where Poly I:C alone produced the largest response (TSPO mRNA in female HP). Despite elevations in TSPO mRNA in females, there were no corresponding changes in protein expression, raising the possibility of sex-dependent differences in post-translational processes for this marker. In the correlation analysis, mRNAs and proteins levels of all three neuroinflammation markers were positively correlated in the overall brain regions of males, but not generalized in females (Suppl. Fig. S2). This suggests that the posttranslational regulation of neuroinflammation markers in females may contribute the resilient outcome of immune regulation.

**Table 3:** Neuroinflammation markers, changes in mRNA and protein levels, and correlation between mRNA and protein expressions.

Sex	Endpoint	Marker	Region	Analysis				*Post-hoc* compared to VV	Protein-mRNA
				Main effect: postnatal		Interaction			
				Statistic (df)	*P* value	Statistic (df)	*P* value		
Male	mRNA	Iba-1	mPFC	F(1,16) = 230.33	<0.0001	F(1,16) = 8.62	0.0097	VL, PL	
			AMY	F(1,16) = 165.08	<0.0001	F(1,16) = 56.41	<0.0001	VL, PL	
			HP	F(1,16) = 593.80	<0.0001	F(1,16) = 38.33	<0.0001	PV, VL, PL	
			TM	F(1,16) = 232.03	<0.0001	F(1,16) = 1.43	*ns*	VL, PL	
		GFAP	mPFC	F(1,16) = 474.33	<0.0001	F(1,16) = 59.83	<0.0001	VL, PL	
			AMY	F(1,16) = 657.53	<0.0001	F(1,16) = 379.92	<0.0001	PV, VL, PL	
			HP	F(1,16) = 917.53	<0.0001	F(1,16) = 13.15	0.0023	VL, PL	
			TM	F(1,16) = 947.50	<0.0001	F(1,16) = 0.01	*ns*	VL, PL	
		TSPO	mPFC	F(1,16) = 868.23	<0.0001	F(1,16) = 81.84	<0.0001	VL, PL	
			AMY	F(1,16) = 733.73	<0.0001	F(1,16) = 364.60	<0.0001	PV, VL, PL	
			HP	F(1,16) = 664.81	<0.0001	F(1,16) = 30.96	<0.0001	PV, VL, PL	
			TM	F(1,16) = 470.88	<0.0001	F(1,16) = 363.57	<0.0001	PL	
Female	mRNA	Iba-1	mPFC	F(1,16) = 227.77	<0.0001	F(1,16) = 0.76	*ns*	PV, VL, PL	
			AMY	F(1,16) = 70.97	<0.0001	F(1,16) = 10.80	0.0046	PV, VL, PL	
			HP	F(1,16) = 4.09	*ns*	F(1,16) = 23.88	0.0002	PV, VL	
			TM	F(1,16) = 50.14	<0.0001	F(1,16) = 0.92	*ns*	VL, PL	
		GFAP	mPFC	F(1,16) = 299.84	<0.0001	F(1,16) = 113.53	<0.0001	PV, VL, PL	
			AMY	F(1,16) = 16.11	0.001	F(1,16) = 0.14	*ns*		
			HP	F(1,16) = 1.72	*ns*	F(1,16) = 0.02	*ns*		
			TM	F(1,16) = 0.08	*ns*	F(1,16) = 0.07	*ns*		
		TSPO	mPFC	F(1,16) = 59.59	<0.0001	F(1,16) = 3.23	*ns*	PV, VL, PL	
			AMY	F(1,16) = 122.66	<0.0001	F(1,16) = 97.23	<0.0001	PV, VL, PL	
			HP	F(1,16) = 7.16	0.0165	F(1,16) = 130.88	<0.0001	PV, VL, PL	
			TM	F(1,16) = 0.01	*ns*	F(1,16) = 2.14	*ns*	PL	
Male	protein	Iba-1	mPFC	F(1,8) = 165.92	<0.0001	F(1,8) = 8.74	0.0182	PV, VL, PL	R^2^ = 0.5748, *P* = 0.0043
			AMY	F(1,8) = 40.10	0.0002	F(1,8) = 34.41	0.0004	PV, VL, PL	R^2^ = 0.7034, *P* = 0.0007
			HP	F(1,8) = 17.97	0.0028	F(1,8) = 5.80	0.0427	VL	R^2^ = 0.5466, *P* = 0.006
			TM	F(1,8) = 108.06	<0.0001	F(1,8) = 0.09	*ns*	VL, PL	R^2^ = 0.5818, *P* = 0.0039
		GFAP	mPFC	F(1,8) = 37.91	0.0003	F(1,8) = 6.12	0.0385	PV, VL, PL	R^2^ = 0.3761, *P* = 0.034
			AMY	F(1,8) = 54.46	<0.0001	F(1,8) = 0.02	*ns*	VL, PL	R^2^ = 0.4205, *P* = 0.0226
			HP	F(1,8) = 53.09	<0.0001	F(1,8) = 8.80	0.018	PL	R^2^ = 0.7659, *P* = 0.0002
			TM	F(1,8) = 65.20	<0.0001	F(1,8) = 28.48	0.0007	PV, VL, PL	R^2^ = 0.7761, *P* = 0.0002
		TSPO	mPFC	F(1,8) = 201.75	<0.0001	F(1,8) = 1.00	*ns*	VL, PL	R^2^ = 0.6421, *P* = 0.0017
			AMY	F(1,8) = 62.67	<0.0001	F(1,8) = 7.66	0.0244	VL, PL	R^2^ = 0.6699, *P* = 0.0011
			HP	F(1,8) = 14.64	0.005	F(1,8) = 0.56	*ns*		R^2^ = 0.2903, *ns*
			TM	F(1,8) = 105.84	<0.0001	F(1,8) = 3.01	*ns*	VL, PL	R^2^ = 0.7567, *P* = 0.0002
Female	protein	Iba-1	mPFC	F(1,8) = 30.53	0.0006	F(1,8) = 0.02	*ns*	VL, PL	R^2^ = 0.3553, *P* = 0.0408
			AMY	F(1,8) = 50.77	<0.0001	F(1,8) = 0.90	*ns*	VL, PL	R^2^ = 0.3117, *ns*
			HP	F(1,8) = 0.07	*ns*	F(1,8) = 6.09	0.0389		R^2^ = 0.0375, *ns*
			TM	F(1,8) = 67.45	<0.0001	F(1,8) = 0.78	*ns*	VL, PL	R^2^ = 0.6582, *P* = 0.0014
		GFAP	mPFC	F(1,8) = 42.48	0.0002	F(1,8) = 33.15	0.0004	PL	R^2^ = 0.2036, *ns*
			AMY	F(1,8) = 8.95	0.0173	F(1,8) = 0.25	*ns*		R^2^ = 0.334, *P* = 0.0491
			HP	F(1,8) = 246.59	<0.0001	F(1,8) = 5.03	*ns*	VL, PL	R^2^ = 0.0032, *ns*
			TM	F(1,8) = 0.00	*ns*	F(1,8) = 0.04	*ns*		R^2^ = 0.0032, *ns*
		TSPO	mPFC	F(1,8) = 2.07	*ns*	F(1,8) = 0.66	*ns*		R^2^ = 0.1452, *ns*
			AMY	F(1,8) = 2.61	*ns*	F(1,8) = 2.08	*ns*		R^2^ = 0.2999, *ns*
			HP	F(1,8) = 0.14	*ns*	F(1,8) = 6.04	0.0394		R^2^ = 0.4028, *P* = 0.0266
			TM	F(1,8) = 0.10	*ns*	F(1,8) = 0.24	*ns*		R^2^ = 0.1575, *ns*

Statistical analysis of neuroinflammatory mRNA and protein markers by brain region for males and females. *ns*, not significant; VV, Vehicle-Vehicle; PV, Poly I:C-Vehicle; VL, Vehicle-LPS; PL, Poly I:C-LPS.

### Expression of anti-inflammatory factors

**Figure 11:**
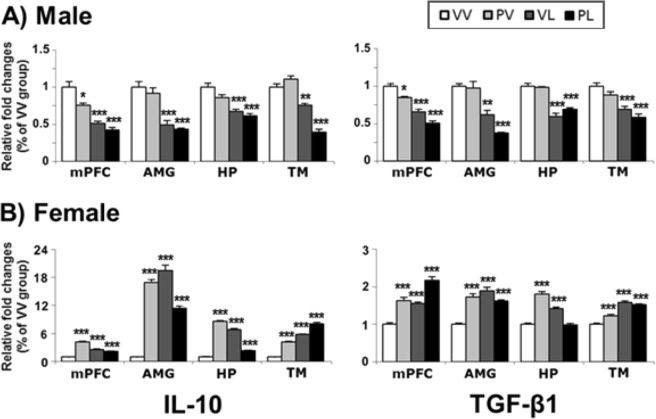
Levels of mRNA expression for anti-inflammatory markers in the brains of (**A**) male and (**B**) female mice following perinatal immune activation, expressed as Mean + SEM (*n* = 5/group) of duplicate experiments. *P < 0.05, **P < 0.01, ***P < 0.001 compared to VV, Bonferroni’s *post-hoc* test. IL-10, interleukin 10; TGF-β1, transforming growth factor beta 1.

**Figure 12:**
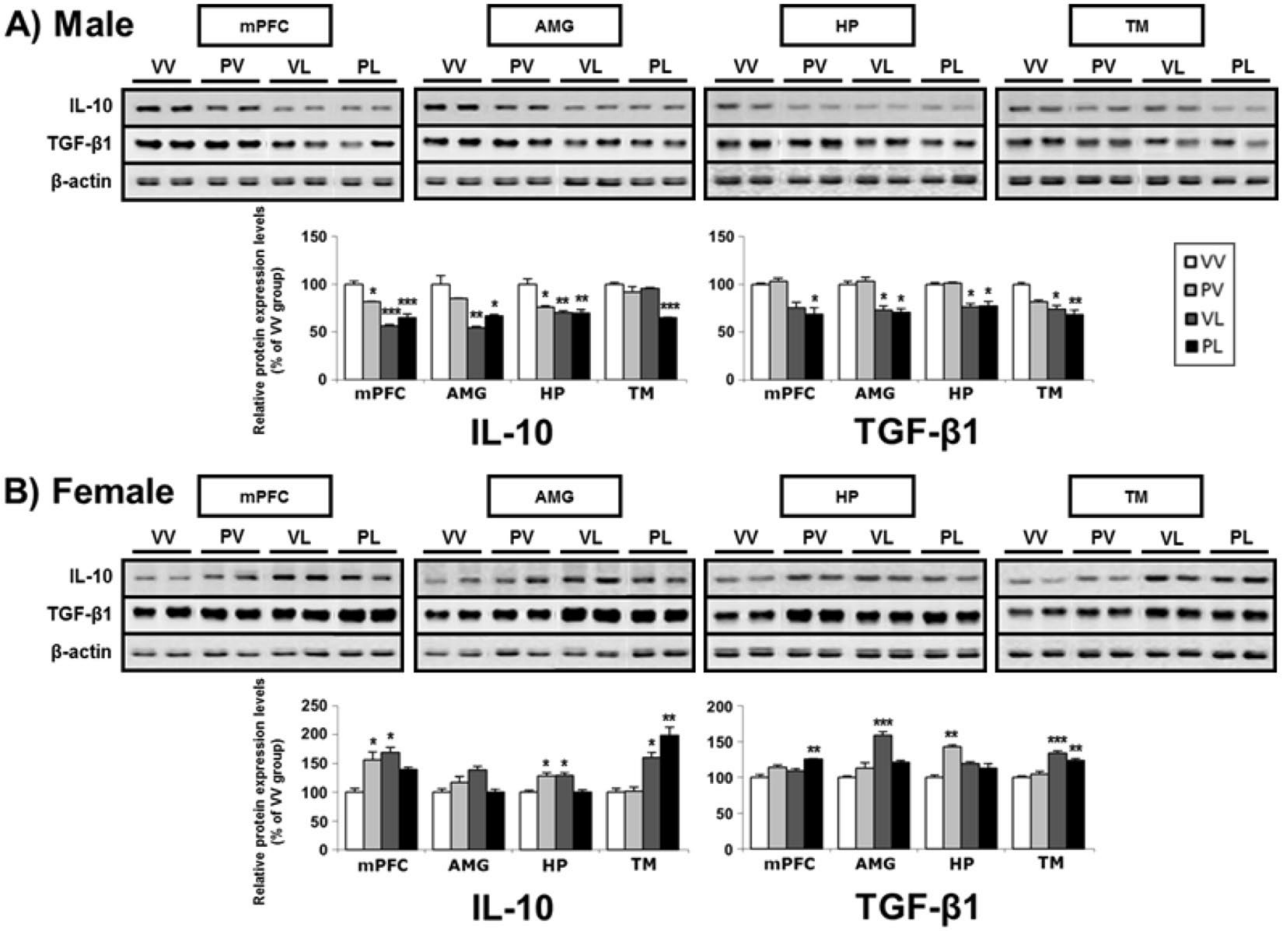
Representative western blots and levels of protein expression for anti-inflammatory markers (and β-actin control) in the brains of (**A**) male and (**B**) female mice following perinatal immune activation, expressed as Mean + SEM (*n* = 3/group) of triplicate experiments. Images of western blots are cropped selections from full length western blots found in supplementary information. *P < 0.05, **P < 0.01, ***P < 0.001 compared to VV, Bonferroni’s *post-hoc* test.

Finally, we examined changes in mRNA and protein levels of the anti-inflammatory factors, interleukin-10 (IL-10) and transforming growth factor beta 1 (TGF-β1) (Table 4). In males, both prenatal and early postnatal immune activation produced decreases in mRNA levels across the regions studied, and the largest effects tended to be in mice that had been exposed to LPS alone or in combination with Poly I:C (Fig. 11). Remarkably, these treatments produced the opposite effects in females, producing increases in mRNA levels of these factors, with all treatments causing the same qualitative effects (Fig. 12). These changes in mRNA levels were accompanied by corresponding changes in protein expression as shown in the correlation analysis (Suppl. Fig. S3), following the same general patterns of decreases and increases respectively. These data indicate that males and females mount dramatically different—virtually opposite—anti-inflammatory responses following perinatal activation of TLR3 and/or TLR4 receptors.

**Table 4:** Anti-inflammatory markers, changes in mRNA and protein levels, and correlation between mRNA and protein expressions.

Sex	Endpoint	Marker	Region	Analysis				*Post-hoc* compared to VV	Protein-mRNA
				Main effect: postnatal		Interaction			
				Statistic (df)	*P* value	Statistic (df)	*P* value		
Male	mRNA	IL-10	mPFC	F(1,16) = 79.79	<0.0001	F(1,16) = 2.86	*ns*	PV, VL, PL	
			AMY	F(1,16) = 70.56	<0.0001	F(1,16) = 0.05	*ns*	VL, PL	
			HP	F(1,16) = 55.94	<0.0001	F(1,16) = 1.16	*ns*	VL, PL	
			TM	F(1,16) = 161.34	<0.0001	F(1,16) = 38.84	<0.0001	VL, PL	
		TGF-β1	mPFC	F(1,16) = 141.11	<0.0001	F(1,16) = 0.00	*ns*	PV, VL, PL	
			AMY	F(1,16) = 77.42	<0.0001	F(1,16) = 3.92	*ns*	VL, PL	
			HP	F(1,16) = 123.40	<0.0001	F(1,16) = 3.23	*ns*	VL, PL	
			TM	F(1,16) = 50.82	<0.0001	F(1,16) = 0.02	*ns*	VL, PL	
Female	mRNA	IL-10	mPFC	F(1,16) = 3.69	*ns*	F(1,16) = 202.28	<0.0001	PV, VL, PL	
			AMY	F(1,16) = 88.10	<0.0001	F(1,16) = 300.38	<0.0001	PV, VL, PL	
			HP	F(1,16) = 1.89	*ns*	F(1,16) = 1510.63	<0.0001	PV, VL, PL	
			TM	F(1,16) = 643.83	<0.0001	F(1,16) = 8.26	0.011	PV, VL, PL	
		TGF-β1	mPFC	F(1,16) = 58.11	<0.0001	F(1,16) = 0.01	*ns*	PV, VL, PL	
			AMY	F(1,16) = 33.59	<0.0001	F(1,16) = 55.28	<0.0001	PV, VL, PL	
			HP	F(1,16) = 17.42	0.0007	F(1,16) = 165.53	<0.0001	PV, VL	
			TM	F(1,16) = 187.92	<0.0001	F(1,16) = 19.47	0.0004	PV, VL, PL	
Male	protein	IL-10	mPFC	F(1,8) = 82.58	<0.0001	F(1,8) = 17.11	0.0033	PV, VL, PL	R^2^ = 0.7171, *P* = 0.0005
			AMY	F(1,8) = 30.02	0.0006	F(1,8) = 5.63	0.045	VL, PL	R^2^ = 0.6021, *P* = 0.003
			HP	F(1,8) = 16.14	0.0039	F(1,8) = 7.16	0.0281	PV, VL, PL	R^2^ = 0.6418, *P* = 0.0017
			TM	F(1,8) = 19.42	0.0023	F(1,8) = 10.54	0.0118	PL	R^2^ = 0.433, *P* = 0.02
		TGF-β1	mPFC	F(1,8) = 25.87	0.0009	F(1,8) = 0.75	*ns*	PL	R^2^ = 0.548, *P* = 0.0059
			AMY	F(1,8) = 36.91	0.0003	f(1,8) = 0.34	*ns*	VL, PL	R^2^ = 0.6755, *P* = 0.001
			HP	F(1,8) = 40.91	0.0002	F(1,8) = 0.00	*ns*	VL, PL	R^2^ = 0.2955, *ns*
			TM	F(1,8) = 24.83	0.0011	F(1,8) = 2.30	*ns*	VL, PL	R^2^ = 0.3307, *P* = 0.0505
Female	protein	IL-10	mPFC	F(1,8) = 6.20	0.0376	F(1,8) = 16.49	0.0036	PV, VL	R^2^ = 0.4854, *P* = 0.0118
			AMY	F(1,8) = 1.52	*ns*	F(1,8) = 9.75	0.0142		R^2^ = 0.3111, *ns*
			HP	F(1,8) = 0.02	*ns*	F(1,8) = 24.18	0.0012	PV, VL	R^2^ = 0.4811, *P* = 0.0124
			TM	F(1,8) = 42.89	0.0002	F(1,8) = 2.33	*ns*	VL, PL	R^2^ = 0.6289, *P* = 0.0021
		TGF-β1	mPFC	F(1,8) = 6.95	0.0299	F(1,8) = 0.05	*ns*	PL	R^2^ = 0.5465, *P* = 0.006
			AMY	F(1,8) = 34.02	0.0004	F(1,8) = 19.17	0.0024	VL	R^2^ = 0.2629, *ns*
			HP	F(1,8) = 1.15	*ns*	F(1,8) = 26.56	0.0009	PV	R^2^ = 0.4865, *P* = 0.0141
			TM	F(1,8) = 55.57	<0.0001	F(1,8) = 4.14	*ns*	VL, PL	R^2^ = 0.7558, *P* = 0.0002

Statistical analysis of anti-inflammatory mRNA and protein markers by brain region for males and females. *ns*, not significant; VV, Vehicle-Vehicle; PV, Poly I:C-Vehicle; VL, Vehicle-LPS; PL, Poly I:C-LPS.

**Figure 13:**
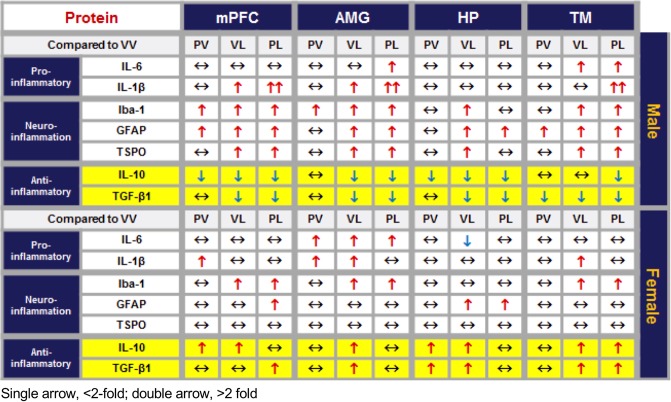
Summary of changes in expression of pro-inflammatory, neuroinflammation, and anti-inflammatory proteins across brain areas and sex. Expressed as relative to VV control; single arrow, <2-fold; double arrow, >2-fold; two-headed arrow, no change.

Qualitative sex-related similarities and differences among the various pro-inflammatory, neuroinflammatory, and anti-inflammatory proteins studied are summarized in Fig. 13.

## Discussion

Transient episodes of EIA can trigger persistent behavioral and molecular adaptations in mice. It is important to emphasize that many of these changes—particularly the molecular changes—were evident long after the acute immune response was triggered. With respect to behavioral endpoints, MIA (Poly I:C administered on E12.5) tended to have minimal effects, whereas PIA (LPS administered on PND9) produced more pronounced effects. In most—though not all—cases, the most dramatic effects were seen with the “two-hit” (combined) treatment (prenatal Poly I:C plus postnatal LPS). Likewise, there were sex differences across some—though not all—behavioral domains, with males being more reliably and robustly affected. With respect to molecular endpoints, we examined changes in mRNA and protein levels for inflammation-related genes (e.g., pro-inflammatory, neuroinflammation, and anti-inflammatory markers) in mice that had received behavioral screening and found both qualitative and quantitative differences. The most striking finding was evidence for fundamental sex-related differences in the type of immune response triggered by perinatal immune activation. We found that both males and females showed significant increases in the expression of pro-inflammatory and neuroinflammation markers in the brain, and that the changes tended to be larger in male mice across all areas examined (mPFC, AMG, HP, and TM). However, we discovered that males and females showed opposite patterns of changes in anti-inflammatory markers: specifically, both IL-10 and TGF-β1 were down-regulated by perinatal immune activation in males, whereas these same markers were significantly up-regulated in females. Our findings suggest that immune system activation during critical periods of development is sufficient to produce persistent alterations in behavior and brain biology—even in the absence of accompanying genetic vulnerability or injury—and that females may mount anti-inflammatory responses that may explain reduced severity of the phenotype in mice of this sex.

### Behavioral changes

MIA on its own produced few effects on behaviors that resemble the core features of ASD in either sex. This outcome was surprising because some of the seminal reports on EIA^13,14^ described profound ASD-like phenotypes in tests quantifying social behavior, ultrasonic vocalizations, and repetitive behaviors. We have also shown negligible effects of prenatal Poly I:C alone on behaviors such as time in the center of an open field^17^, and sleep and epileptiform activity^11^. Recent work suggests that gut bacterial flora, which can differ among commercial suppliers and institutional animal care facilities, plays a critical role in the ability of prenatal Poly I:C to produce behavioral effects. Indeed, we purchased our mice from a supplier (Jackson Labs) that lack a commensal segmented filamentous bacteria in the gut that cause induction of an immune factor (IL-17a) that regulates sensitivity to the Poly I:C^18,32^, providing a potential explanation for our lack of strong effects. Alternatively, the lack of reliable Poly I:C effects could also be related to batch variability; indeed, we used multiple batches of Poly I:C that might conceivably have differences in potency or efficacy^32^. In contrast, PIA tended to be sufficient to produce signs of the 3 core features of ASD. Whereas communication-like behavior was affected in both sexes—with virtually identical effects on the numbers, patterns, and durations of USVs—changes in social behavior in a social approach test^25^ were observed only in males, with no effects whatsoever in females. In addition, LPS treatment produced strong effects in the social scent test, which has been validated only in males^24^. Male mice treated with LPS only also showed enhanced performance on the rotarod; while this test is often used to quantify motor performance capabilities, there is precedent in the literature for interpreting it as an indicator of repetitive or restricted movements^31^. The behavioral effects were generally strongest with the two-hit combination, although in the case of rotarod behavior, the early Poly I:C effect appeared to have a protective-like effect that was unexpected and is difficult to explain. Finally, males showed larger increases in anxiety-like behavior—which is often a characteristic of ASD in humans^33^ despite not being a diagnostic criterion for the condition—in males but not females, without accompanying non-specific alterations in locomotor activity that might complicate data interpretation. These patterns of behavioral changes, together with their similar ability to produce alterations in sleep and epileptiform behavior^11^—which are frequently co-morbid with ASD—provide support for this model in the context of ASD research. In addition, the fact that we found in the behavioral tests that males are more frequently affected than females is consistent with the higher prevalence of ASD in males (4:1), and suggests sex-dependent differences in the distribution of traits regulating behavior.

### Molecular changes

EIA produced elevations in pro-inflammatory (TNFα, iNOS, IL-6, and IL-1β) and neuroinflammation markers (Iba-1, GFAP, and TSPO). Effects were observed in both sexes; while the effects tended to be larger in males than in females, these studies were intended to yield a qualitative “first pass” analysis of the existence of potential sex differences on a large number of markers rather than a rigorous quantitative comparison of a more restricted list. Qualitatively similar effects were seen with mRNA and protein, although increases in mRNA levels in males were on occasion considerably higher than those seen in females despite the similarities in levels of protein, raising the possibility of post-translational effects that limit the scale of changes in protein expression. Such drastic increases in innate immune mRNA, with relatively smaller increases in protein levels parallel similar findings in microglia that are the result of translational suppression mechanisms^34^. There was a small number of specific cases where increases in mRNA levels of pro-inflammatory/neuroinflammation markers were not accompanied by changes in protein expression, also suggesting post-translational processes that further regulate protein expression. These individual cases in specific brain regions do not detract from the significance of the often massive changes in both mRNA and protein levels in other brain areas. Our findings in brain are broadly consistent with the existing literature. Perinatal immune activation in mice also increases TNFα, IL-6 and IL-1β mRNA levels in placenta^35,36^, and single maternal injection of IL-6 resulted in ASD-like signs in the offspring^13^. Interestingly, male mouse pup-derived astrocyte cultures show markedly higher expression of TNFα, IL-6 and IL-1β mRNA in response to LPS compared to those derived from females^37^. Collectively, the present studies are consistent with data from humans. Altered inflammatory cytokine levels in postmortem brain samples and serum plasma samples from individuals with ASDs have been previously reported in numerous studies. For example, pro-inflammatory cytokines such as TNFα, IL-6 and IL-1β are reportedly elevated^38–40^. In addition, TLR3 and TLR4 activation upregulates cytokine expression in peripheral blood monocyte cultures from children with ASD^41^, although sex differences were not examined. Similarly, while sex differences were not examined, neuroinflammation markers are also elevated in individuals with ASD^42–48^.

Although we found the direction of changes of pro-inflammatory markers were qualitatively similar in male and female mice, there were major sex differences for anti-inflammatory markers. Perinatal immune activation produced decreases in mRNA and protein levels of IL-10 and TGF-β1 in males, but increases in the same markers in females. There is little evidence in the literature that such differences have been previously studied or described. LPS produces decreases in IL-10 levels in macrophage cultures from BTBR T + tf/J (BTBR) mice, which show ASD-like features^49^. Interestingly, female rats had significantly higher levels of mRNA for IL-10 and its receptor IL-10ra than males in the cortex and HP during normal development (at P0, P4 and P60)^50^. Anti-inflammatory cytokines such as IL-10 and TGF-β1 are decreased in serum from individuals with ASD^51–53^, although sex differences were not examined. The molecular mechanisms underlying sex-different gene regulation by perinatal immune activations are currently unknown, but it is likely that genes linked to sex chromosomes, hormonal changes, and/or their interactions may underlie these differences^54^. The fact that Poly I:C and/or LPS both produced changes in these markers, whereas Poly I:C consistently produced negligible effects on behavior, suggests that molecular techniques have a sufficiently high sensitivity to detect effects that are below the threshold needed to significantly alter behavior, as was seen previously in electrophysiology studies^17^. Regardless, our findings of sex differences in anti-inflammatory responses raise the possibility of protective (resilience-related) processes that reduce the prevalence of ASD in females.

### Limitations of the paradigms

Our studies characterized the effects of using Poly I:C as the trigger for MIA and LPS as the trigger for PIA. We used this treatment order because of evidence showing that TLR3 expression is high but steadily decreasing during prenatal development, whereas TLR4 expression is low but steadily increasing over the same period^55^. Postnatal LPS administration also models the high risk of exposure to bacterial infections occurring during this period of development. PND9 in a mouse approximates neurodevelopmental milestones—including brain growth, gliogenesis, and increases in axonal and dendritic density—present in humans at or slightly before, full-term birth^56^. In humans, labor produces over a 10-fold increase in microbial invasion of the amniotic cavity (MIAC)^57,58^, and pre-term birth is highly associated with MIAC and is a risk factor for ASD^58,59^. Furthermore, neonates are at high risk for bacterial infections^60^. Other permutations of this treatment regimen, or experiments in which gene X environment interactions are examined, may yield different outcomes. There are several important caveats with our measurements of immune markers in the brain. Because rapid dissection was used, we cannot exclude peripheral blood as an important source for the cytokines detected here. However, changes in astrocyte and microglia markers support inflammatory changes in the central nervous system in this model. Additionally, many of these cytokine markers are normally found at low levels in the brain, which while allowing accurate detection of qualitative elevations from baseline can lead to imprecision in the measurement of baseline levels or the magnitude of change.

### Implications

These findings provide support for the utility of MIA, PIA, and combined treatment for the study of ASDs on several levels. First, the patterns of behavioral changes, together with their prevalence in males, fit well with the condition as it occurs in humans. The observation that both males and females mount persistent pro-inflammatory responses, whereas only females mount accompanying anti-inflammatory responses, represents a new and potentially important discovery that might aid in understanding sex differences in prevalence and tailoring new treatments. Together, these findings add to accumulating evidence for an immune subtype of ASD^7,61^, and suggest that immune-related pathophysiologies can differ between sexes. The ability to better identify subtypes of this syndrome will facilitate care and perhaps provide a justification for investigations into treatment strategies (e.g., immune-modulating agents) that might not otherwise be considered for ASDs.

## Supplementary information

Supplemental Figures

## Data Availability

The datasets generated during and/or analysed during the current study are available from the corresponding author on reasonable request.
