# Supplementary material for: Maternal and early postnatal immune activation produce sex-specific effects on autism-like behaviors and neuroimmune function in mice
Source: Sci Rep. 2019 Nov 15;9:16928. doi: 10.1038/s41598-019-53294-z (PMC6858355; doi:10.1038/s41598-019-53294-z)

Supplementary Information

Maternal and early postnatal immune activation produce sex-specific effects on autism-like behaviors and neuroimmune function in mice.

William A. Carlezon Jr, Woori Kim, Galen Missig, Beate C. Finger, Samantha M. Landino, Abigail J. Alexander, Emery L. Mokler, James O. Robbins, Yan Li, Vadim Y. Bolshakov, Christopher J. McDougle, Kwang-Soo Kim

Fig. S1 Correlation between mRNA and protein expression levels of pro-inflammatory markers (**A**) IL-6 and (**B**) IL-1β in the brains of male and female mice. Pearson linear correlation coefficients R^2 and their *P* values are indicated in the graph.

Fig. S2 Correlation between mRNA and protein expression levels of neuroinflammation markers (**A**) Iba-1, (**B**) GFAP and (**C**) TSPO in the brains of male and female mice. Pearson linear correlation coefficients R^2 and their *P* values are indicated in the graph.

Fig. S3 Correlation between mRNA and protein expression levels of anti-inflammatory markers (**A**) IL-10 and (**B**) TGF-β1 in the brains of male and female mice. Pearson linear correlation coefficients R^2 and their *P* values are indicated in the graph.

Fig. S4 Full length western blots for each of the proteins analyzed including 1) IL-6, 2) IBA-1, 3) GFAP, 4) TSPO, 5), IL-10, 6) TGF-β1, 7) β-actin. Red selection indicates the representative western blots displayed in **Fig. 8,10,12**.

Supplemental Figure S1

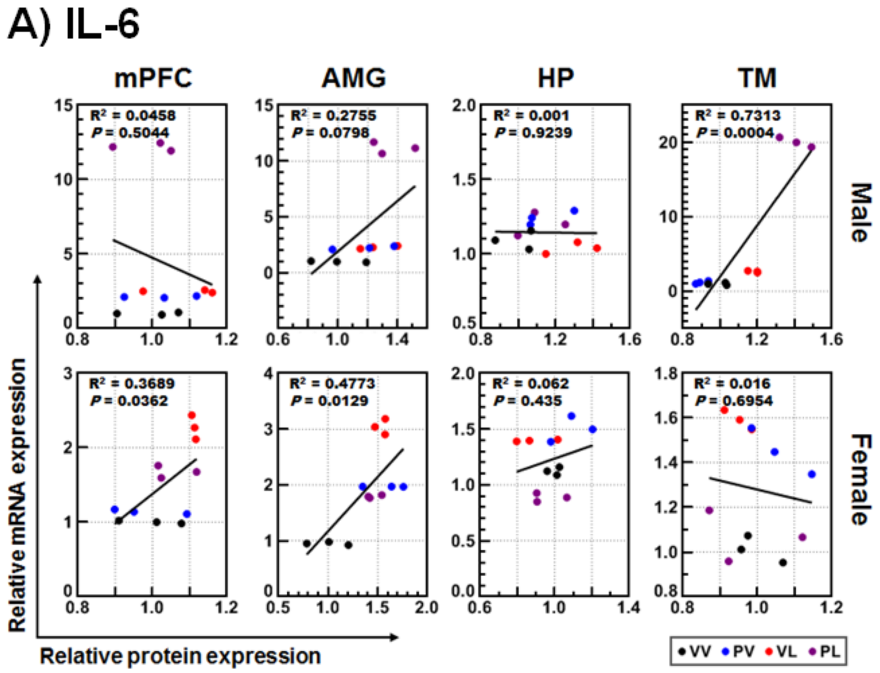

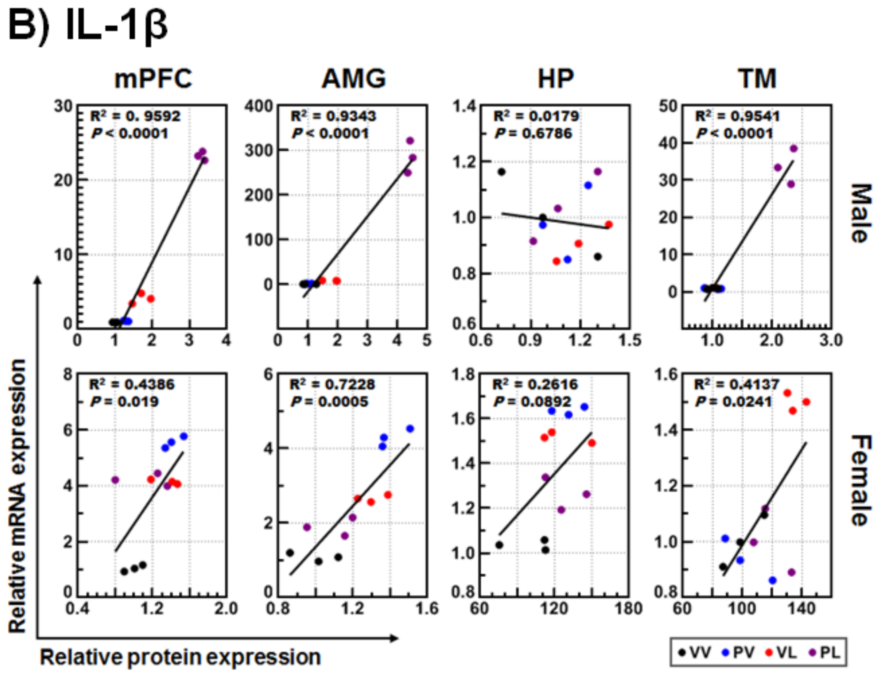

Supplemental Figure S2

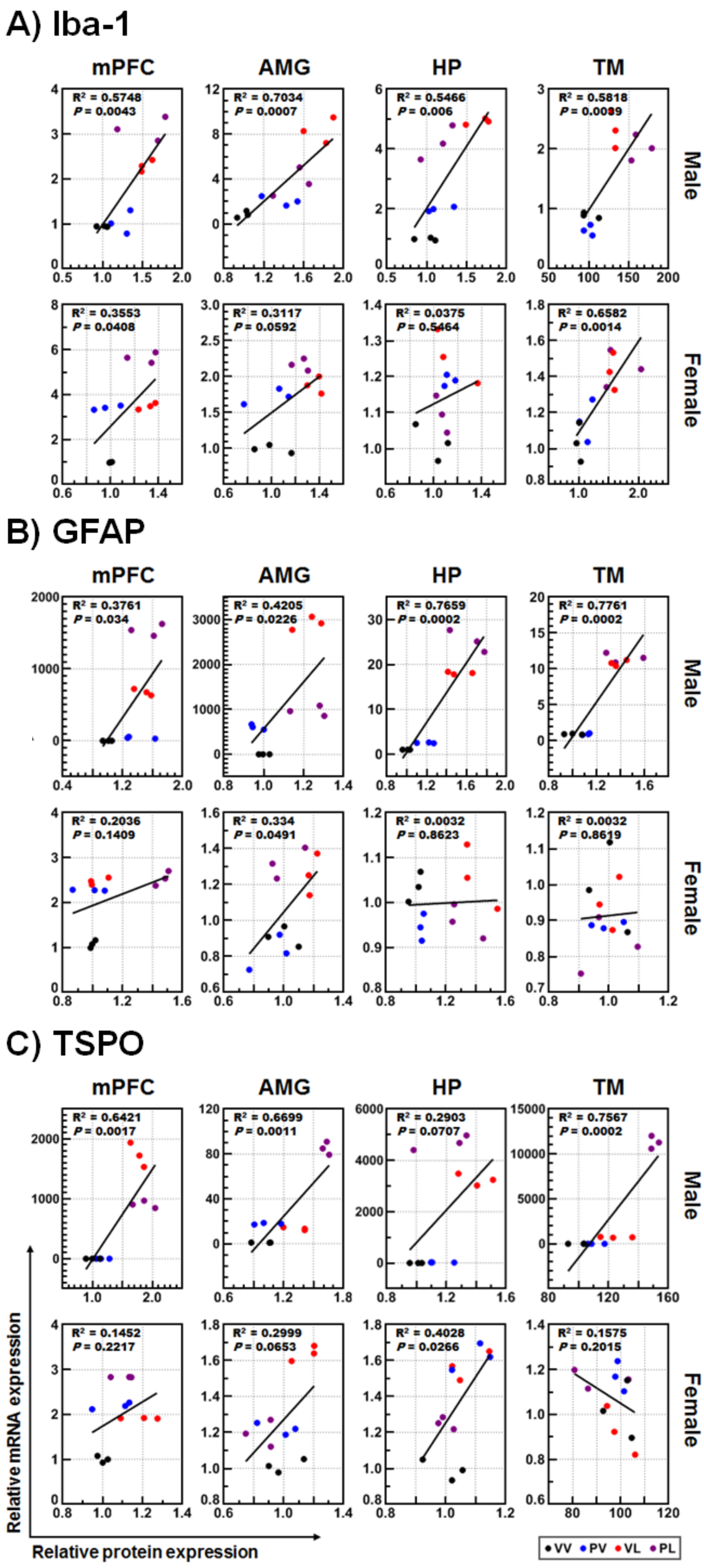

Supplemental Figure S3

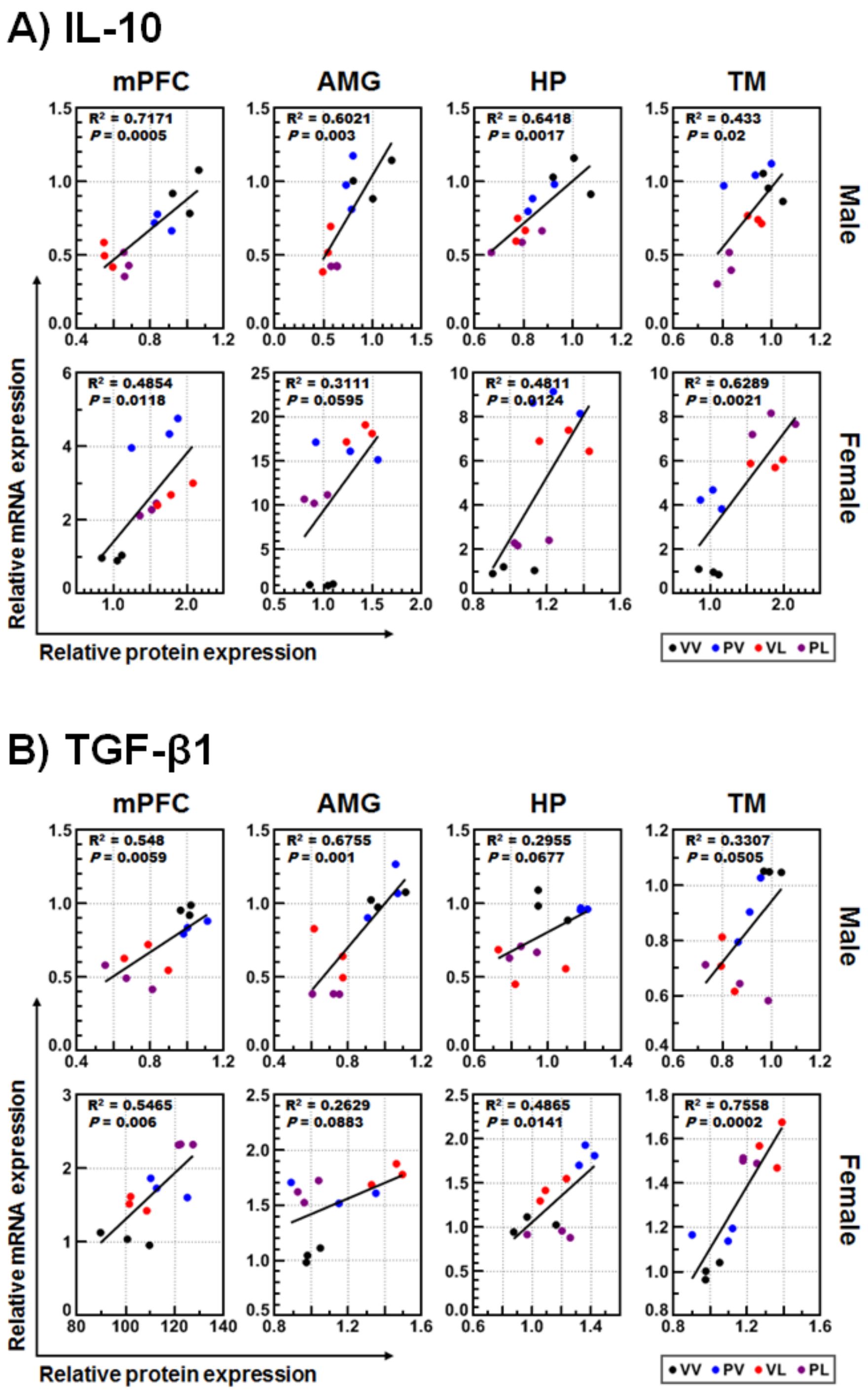

1. IL-6

Male

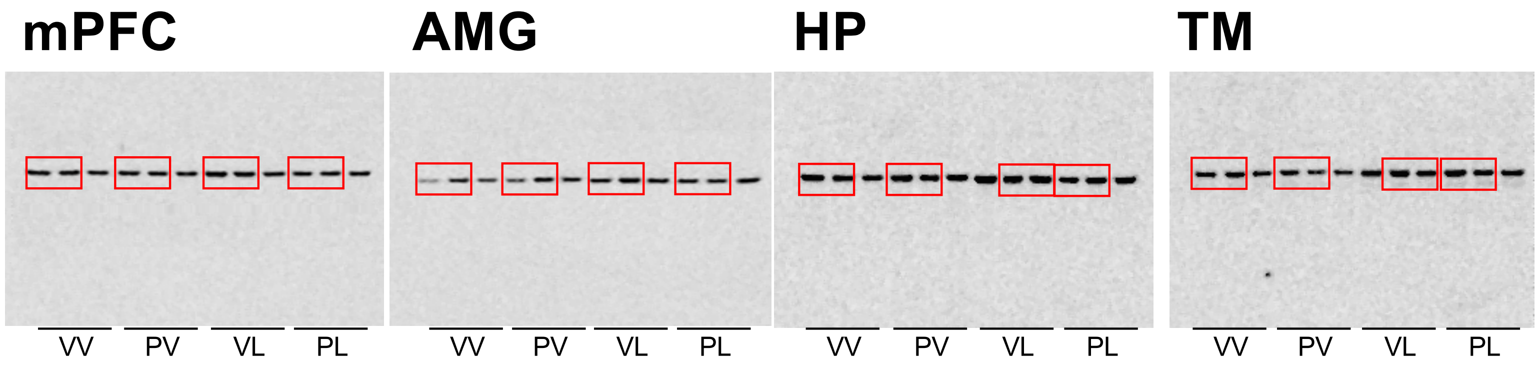

Female

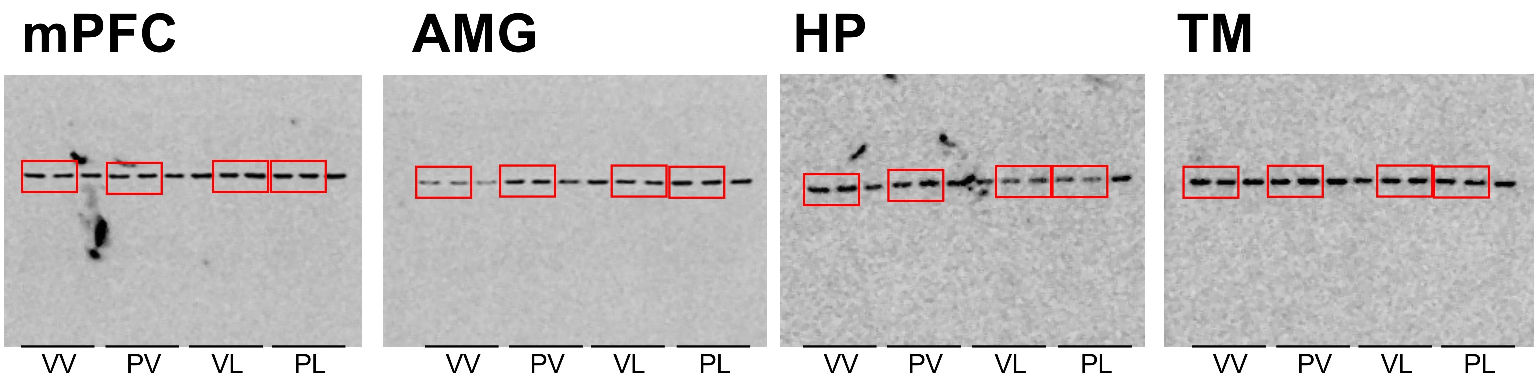

2. Iba-1

Male

mPFC AMG HP TM

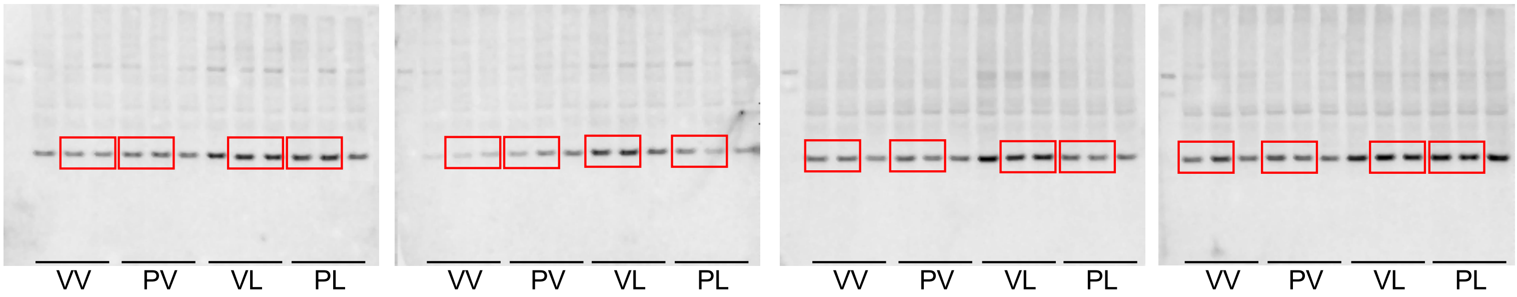

Female

mPFC AMG HP TM

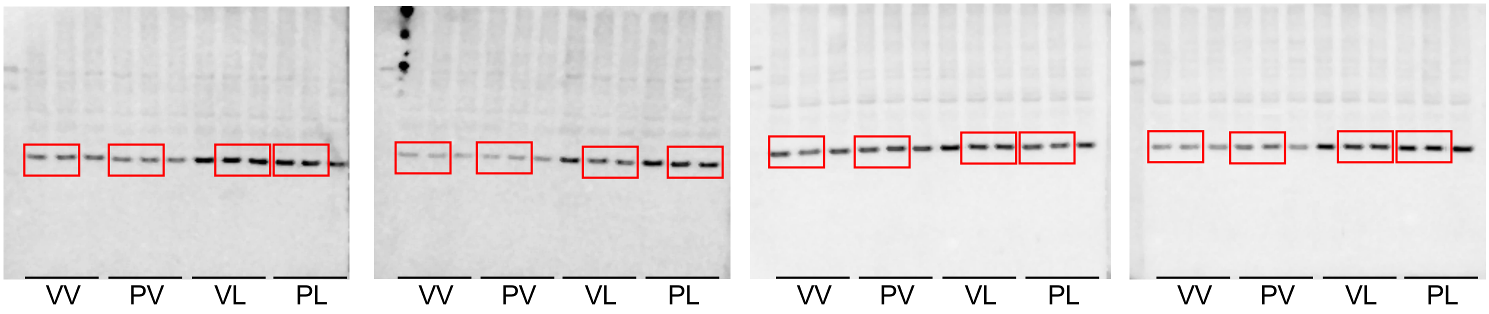

3. GFAP

Male

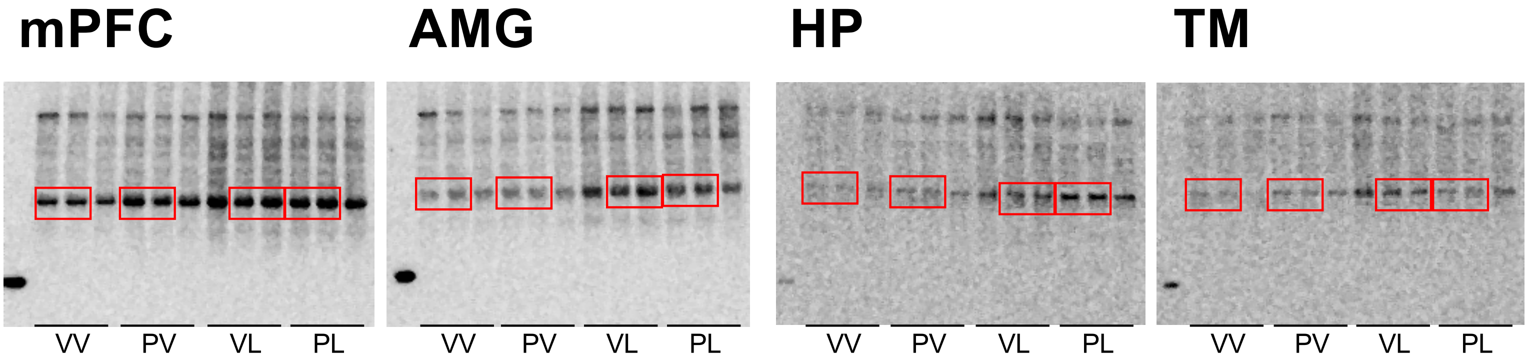

Female

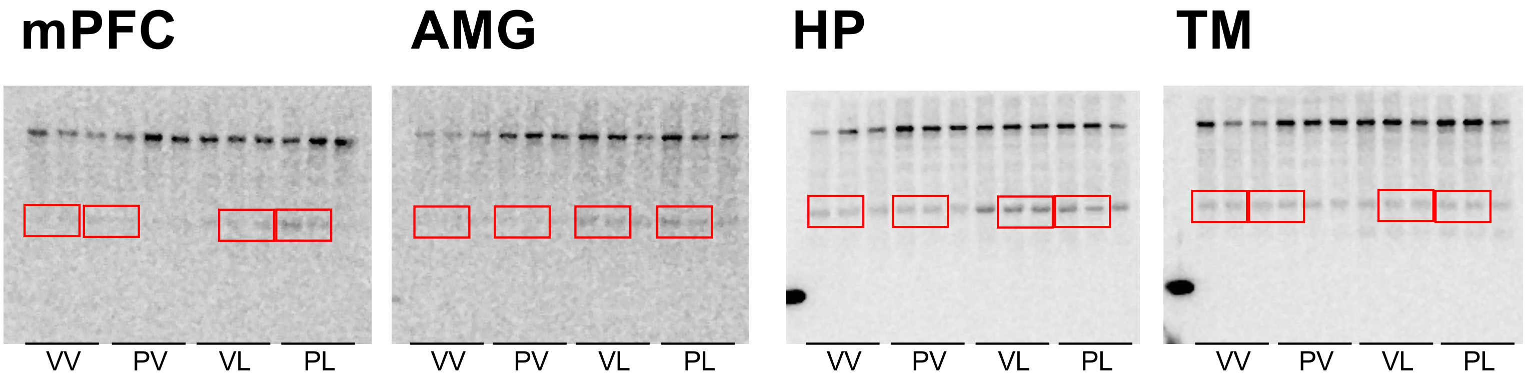

4. TSPO

Male

mPFC

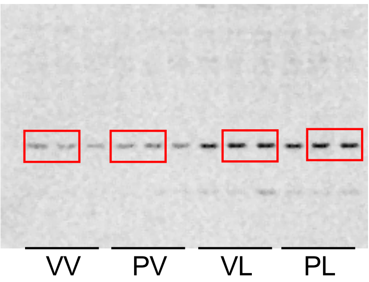

AMG

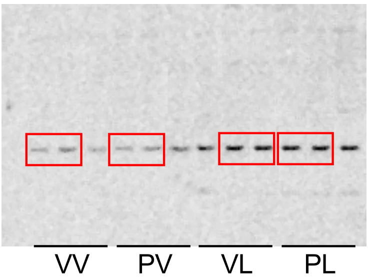

HP

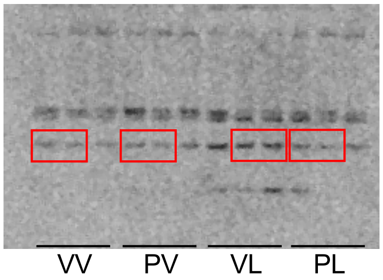

TM

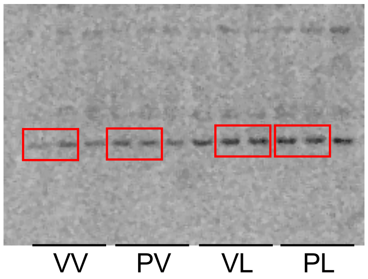

Female

mPFC

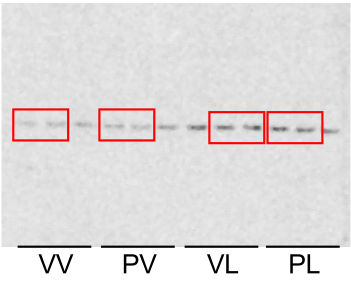

AMG

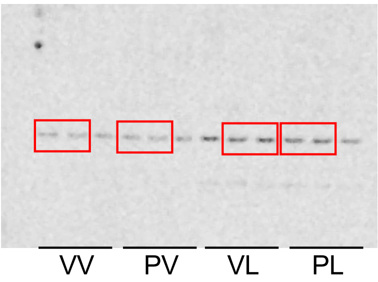

HP

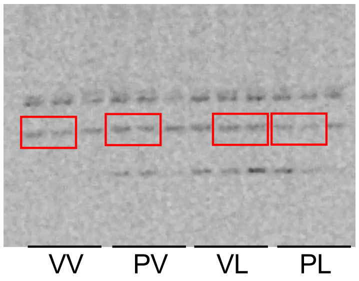

TM

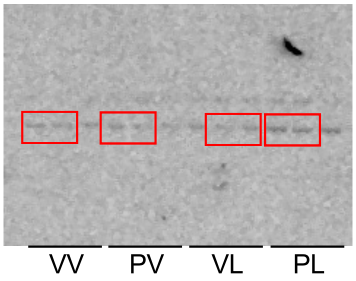

5. IL-10

Male

mPFC

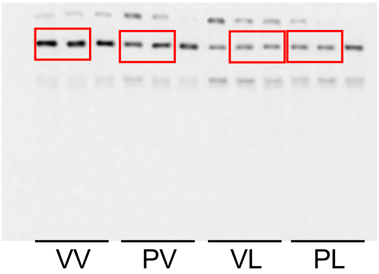

AMG

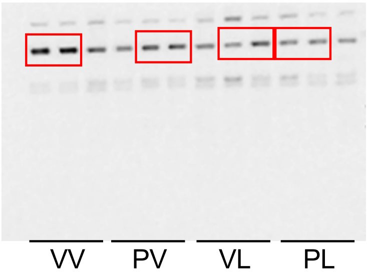

HP

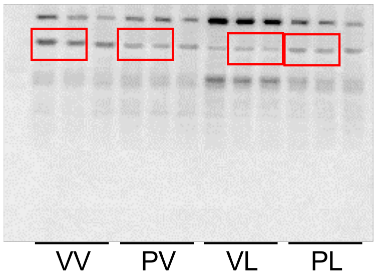

TM

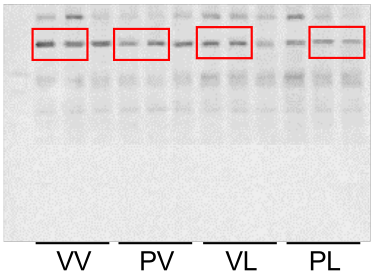

Female

mPFC

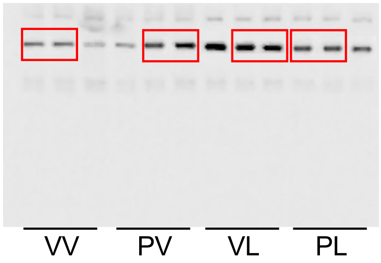

AMG

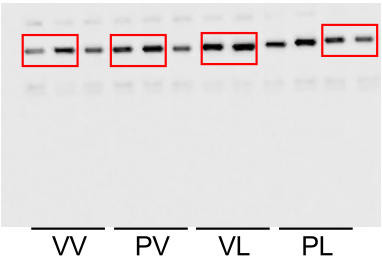

HP

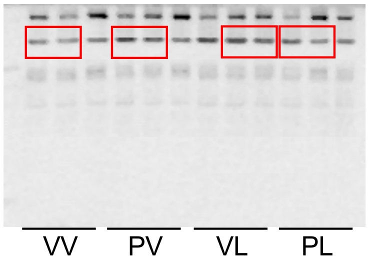

TM

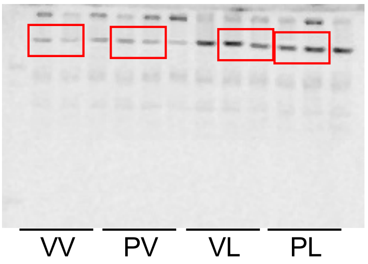

6.TGF-β1

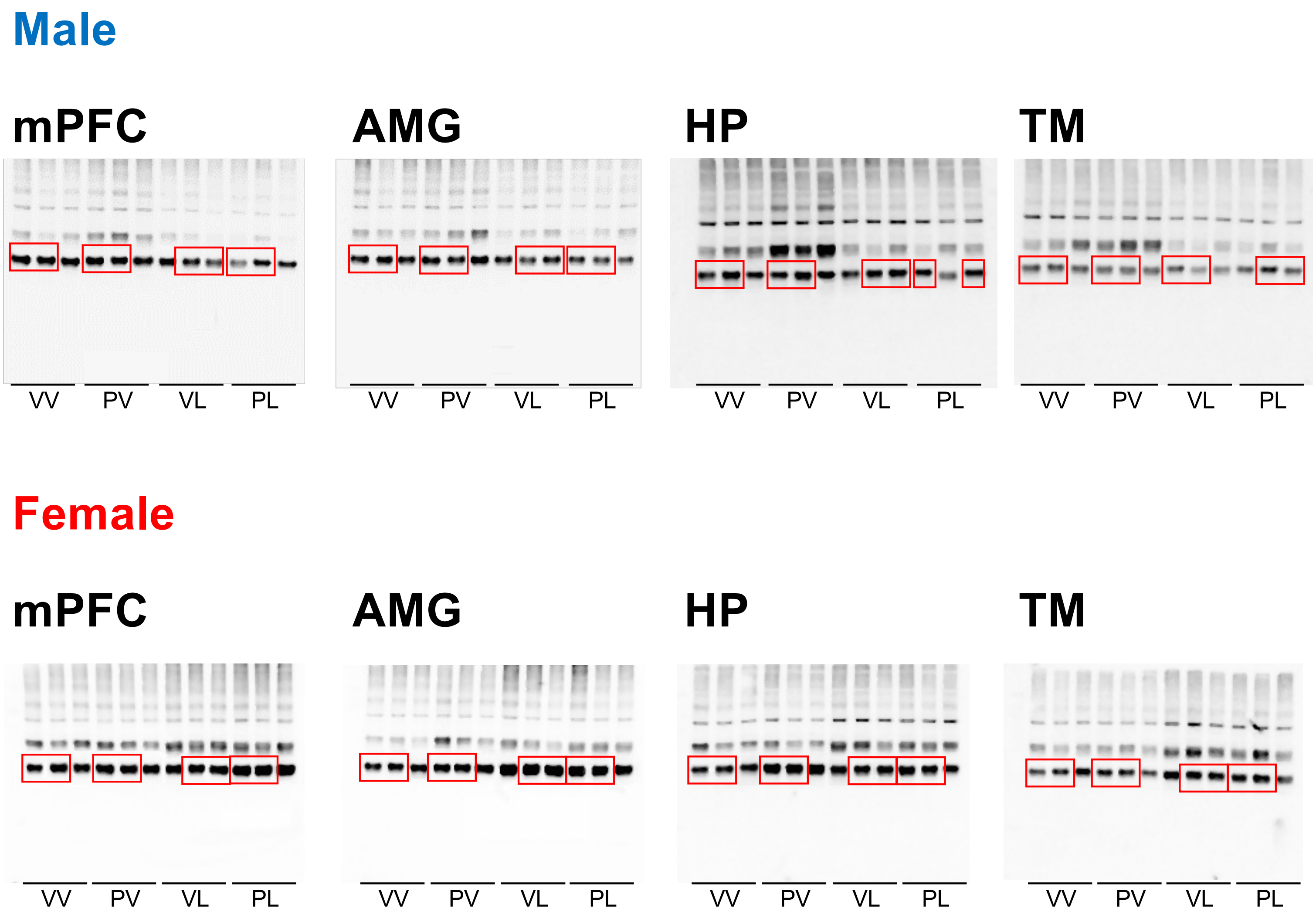
Male
mPFC
AMG
HP
TM
VV PV VL PL
VV PV VL PL
VV PV VL PL
VV PV VL PL
Female
mPFC
AMG
HP
TM
VV PV VL PL
VV PV VL PL
VV PV VL PL
VV PV VL PL

7. β-actin

Male

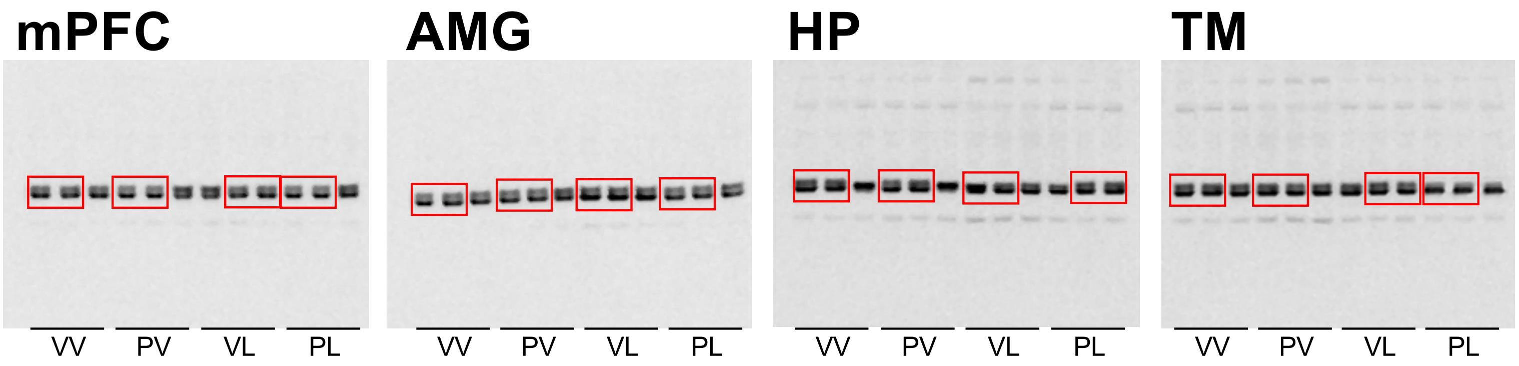

Female

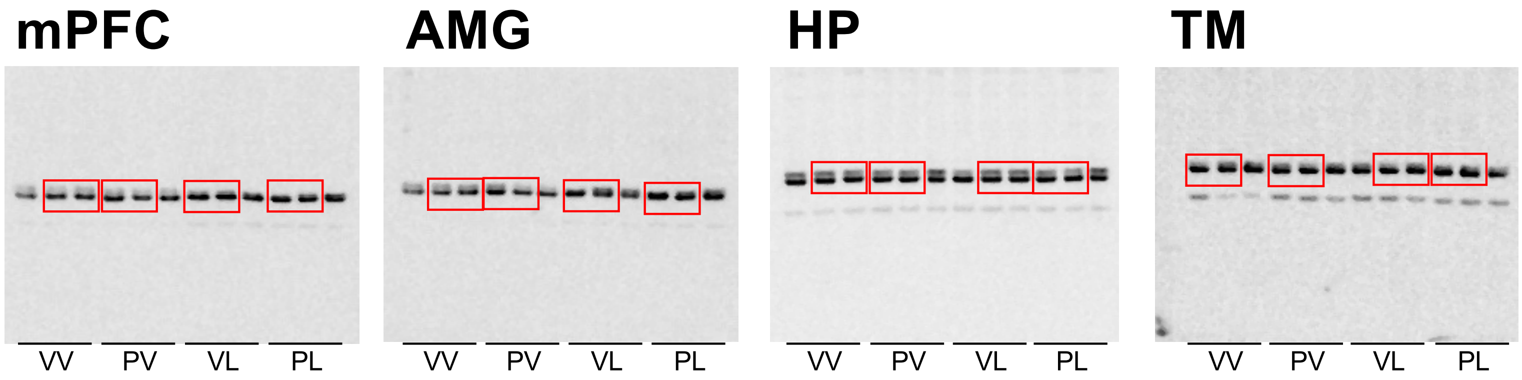

Supplement: Supplementary file 1 — Supplemental Figures [file 41598_2019_53294_MOESM1_ESM.pdf]
